# Infectivity of *Plasmodium* parasites to *Aedes aegypti* and *Anopheles stephensi* mosquitoes maintained on blood-free meals of SkitoSnack

**DOI:** 10.1186/s13071-024-06364-9

**Published:** 2024-07-06

**Authors:** Kristina K. Gonzales-Wartz, Juliana M. Sá, Kevin Lee, Yonas Gebremicale, Bingbing Deng, Carole A. Long, Tales V. Pascini, Andre Laughinghouse, Samuel E. Moretz, Ana M. Ortega-Villa, Michael P. Fay, Thomas E. Wellems

**Affiliations:** 1 Laboratory of Malaria and Vector Research, National Institute of Allergy and Infectious Disease, National Institutes of Health, Bethesda, MD USA; 2 Biostatistics Research Branch, Division of Clinical Research, National Institute of Allergy and Infectious Disease, National Institutes of Health, Bethesda, MD USA

**Keywords:** Insect rearing, Malaria transmission, *Plasmodium falciparum*, *Plasmodium gallinaceum*, Vector-borne disease

## Abstract

**Background:**

*Aedes* and *Anopheles* mosquitoes are responsible for tremendous global health burdens from their transmission of pathogens causing malaria, lymphatic filariasis, dengue, and yellow fever. Innovative vector control strategies will help to reduce the prevalence of these diseases. Mass rearing of mosquitoes for research and support of these strategies presently depends on meals of vertebrate blood, which is subject to acquisition, handling, and storage issues. Various blood-free replacements have been formulated for these mosquitoes, but none of these replacements are in wide use, and little is known about their potential impact on competence of the mosquitoes for *Plasmodium* infection.

**Methods:**

Colonies of *Aedes aegypti* and *Anopheles stephensi* were continuously maintained on a blood-free replacement (SkitoSnack; SS) or bovine blood (BB) and monitored for engorgement and hatch rates. Infections of *Ae. aegypti* and *An. stephensi* were assessed with *Plasmodium gallinaceum* and *P. falciparum*, respectively.

**Results:**

Replicate colonies of mosquitoes were maintained on BB or SS for 10 generations of *Ae. aegypti* and more than 63 generations of *An. stephensi*. The odds of engorgement by SS- relative to BB-maintained mosquitoes were higher for both *Ae. aegypti* (OR = 2.6, 95% CI 1.3–5.2) and *An. stephensi* (OR 2.7, 95% CI 1.4–5.5), while lower odds of hatching were found for eggs from the SS-maintained mosquitoes of both species (*Ae. aegypti* OR = 0.40, 95% CI 0.26–0.62; *An. stephensi* OR = 0.59, 95% CI 0.36–0.96). Oocyst counts were similar for *P. gallinaceum* infections of *Ae. aegypti* mosquitoes maintained on SS or BB (mean ratio = [mean on SS]/[mean on BB] = 1.11, 95% CI 0.85–1.49). Similar oocyst counts were also observed from the *P. falciparum* infections of SS- or BB-maintained *An. stephensi* (mean ratio = 0.76, 95% CI 0.44–1.37). The average counts of sporozoites/mosquito showed no evidence of reductions in the SS-maintained relative to BB-maintained mosquitoes of both species.

**Conclusions:**

*Aedes aegypti* and *An. stephensi* can be reliably maintained on SS over multiple generations and are as competent for *Plasmodium* infection as mosquitoes maintained on BB. Use of SS alleviates the need to acquire and preserve blood for mosquito husbandry and may support new initiatives in fundamental and applied research, including novel manipulations of midgut microbiota and factors important to the mosquito life cycle and pathogen susceptibility.

**Graphical Abstract:**

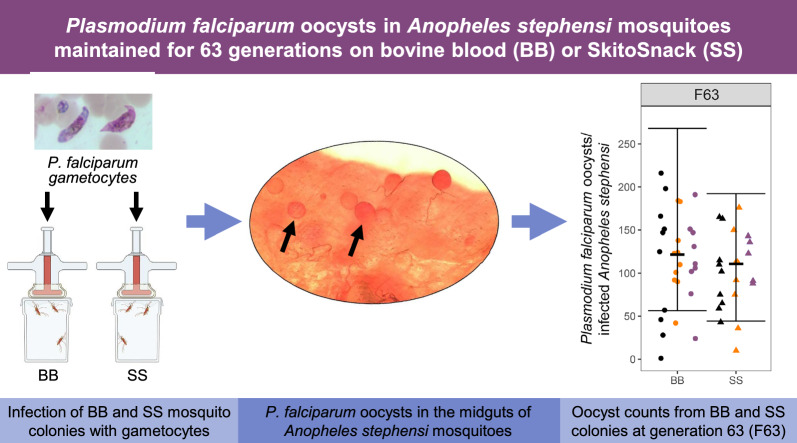

**Supplementary Information:**

The online version contains supplementary material available at 10.1186/s13071-024-06364-9.

## Background

A great range of evolutionarily diverse and highly adaptable mosquito species are associated with the transmission of infectious disease agents, several of which have had profound impacts in human history [1]. Among more than 100 genera and several thousand species of mosquitoes [2], pathogens transmitted by various species of *Aedes* and *Anopheles* have been particularly devastating. *Aedes aegypti*, frequently referred to as the yellow fever mosquito, is not only the main vector for the yellow fever virus, but it is also a major vector for other flaviviruses such as dengue, chikungunya, and Zika fevers [3–6]. Transmission of these pathogens may be facilitated by factors that increase the presence and abundance of mosquito vectors, including effects of ecosystem disruptions, widespread urbanization, and the various impacts of climate warming [7]. *Aedes aegypti* can also transmit *Plasmodium gallinaceum*, which causes an avian malaria that can be highly destructive to flocks of domestic chickens [8].

Human malaria parasites are transmitted by *Anopheles* mosquitoes and caused an estimated 249 million cases and 608,000 deaths in 2022, of which over 90% were in Africa [9, 10]. *Anopheles stephensi* is a major vector of *Plasmodium falciparum* and *P. vivax* in India and Western Asia [11]. This species has spread to Sri Lanka and Saudi Arabia, from Oman to Djibouti and Ethiopia, and has recently been reported in Ghana, raising concern that this expansion may undermine malaria control in Africa [12–16]. New strategies will be needed to counter this expansion as well as the challenges of insecticide resistance [17, 18] and climate change [19] that may compromise the control of mosquito-borne diseases. In one such strategy, *Wolbachia* infections of mosquitoes are being evaluated for their potential to suppress vector populations and combat the transmission of pathogens including arboviruses and *Plasmodium* [20–22].

Research, development, and implementation of new mosquito vector control strategies are supported by mass rearing of mosquitoes. *Aedes* *aegypti* and *An*. *stephensi* are found in two subfamilies of the *Culicidae* (*Culicinae* and *Anophelinae*, respectively) [23]; both mosquito species are anautogenous, requiring a blood meal for egg development and offspring production. Mass rearing thus depends upon the acquisition, handling, and storage of large amounts of blood, which may be obtained from various vertebrates [24–26]. Blood supplies are best used within its recommended shelf life (typically 4 weeks), require cold storage, can present effects from the vertebrate diet and ingested agents, such as drugs, supplements, or antibodies, and, under some circumstances, may risk the introduction of unwanted pathogens into a sterile insectary environment [27–30]. Replacement blood-free meals that satisfy the anautogenous requirement will help to address these issues, avoid animal or human ethics concerns, and promote dependable productivity and vector competence of mosquito colonies.

An early report described investigations of egg production from milk-fed *Ae. aegypti* and *Anopheles quadrimaculatus* mosquitoes, and a meal of proteose-peptone, liver concentrate, and casein hydrolysate was found to support *An. quadrimaculatus* egg viability [31]. These early “blood substitute” meals showed that blood is not an absolute requirement for egg production, but fecundity rates proved to be very low. Improved blood-free meals were subsequently developed for the maintenance of *Ae. aegypti*, *Aedes albopictus*, *Anopheles* mosquitoes, and *Wolbachia*-infected *Ae. aegypti* [27, 32–36].

Much remains to be learned about the vector competence of mosquitoes maintained on such replacements. Recently, no difference was found between the body (thorax and abdomen) and head infection titers of dengue virus serotype 2 in *Ae. aegypti* maintained on bovine blood (BB) or a replacement blood-free meal, SkitoSnack (SS), for 10 or 12 generations; however, relatively lower body infection titers were obtained from SS-maintained mosquitoes infected with dengue virus serotype 4 [37]. Blood meal components are known to stimulate the proliferation of midgut microbiota, which can alter tissue barriers to infection and induce pathways involved in immune protective responses [38]. In view of these findings, artificial meals like SS need to be thoroughly evaluated before they can be routinely used in mosquito insectaries.

In the present study, we have focused on two principal questions:

- (1) Are *Ae. aegypti* mosquitoes maintained on SS or BB susceptible to infection by *P*. *gallinaceum* avian malaria parasites at comparable levels?
- (2) Can SS support *An*. *stephensi* mosquito colonies over multiple continuous generations and maintain their susceptibility to infection by human *P*. *falciparum* malaria parasites, as demonstrated by oocyst and sporozoite development within the mosquitoes?

## Methods

### Source mosquitoes and initial production of colony replicates

Source populations of 500–600 mosquitoes were obtained from *Ae*. *aegypti* Liverpool [4, 39, 40] and *An*. *stephensi* Nijmegen (sda500) [41, 42] stocks maintained in the Laboratory of Malaria and Vector Research (LMVR) Insectary at the National Institute of Allergy and Infectious Diseases (NIAID), National Institutes of Health (NIH), Rockville, MD, USA. Mosquitoes of all life stages were housed inside a walk-in insect environmental chamber (Conviron Controlled Environments Limited, Winnipeg, Manitoba, Canada) at 27 °C, 75% humidity, and a 12 h light/dark cycle. To start each of the individual *Ae*. *aegypti* replicates, approximately 200 dried eggs from the source population were placed into a 8 oz paper cup containing 100 ml distilled water and vacuumed hatched for 30 min. The hatched larvae were transferred to a shallow plastic pan (Cambro 12CW148 Camwear 2.5" deep polycarbonate food pan, Webstaurant Store, Cumberland, MD) with 1 l of 25 °C distilled water. To start each of the individual *An. stephensi* replicates, approximately 200 freshly laid eggs were rinsed and put back into the shallow plastic pan containing 1 l of fresh 25 °C distilled water for hatching. The larvae of both species were maintained with powdered or pelleted Tetramin^®^ Tropical Flakes fish food (Spectrum Brands Pet, LLC, Blacksburg, VA, USA) ad libitum until they reached pupal stage. Pupae were transferred into a small paper cup filled with approximately 200 ml of distilled water and placed inside a 1-gallon mosquito container, which was then closed by a screen at the top. Adults from the pupae were fed through the screen by a cotton wool ball soaked with 10% Karo^®^ dark corn syrup (ACH Food Companies, Inc., Chicago, IL, USA) until preparation for BB or SS feeding (described below).

### SkitoSnack protocol

The powdered ingredients for SS were combined, mixed thoroughly, and stored in a capped plastic Erlenmeyer flask at room temperature (25 °C) as previously described [37] (Additional File 1: Table S1). To prepare the meal, deionized water was added to 0.7 g of powder to the final volume of 3 ml and vortexed for 3–5 min until fully dissolved. The brown-colored meal was pipetted into a glass feeder at 37 °C and offered, within 3 h of the meal preparation, to adult mosquitoes that had been starved for 16 h by replacing the 10% Karo^®^ soaked cotton wool balls with water-soaked ones.

### Bovine blood- and SkitoSnack-maintained mosquito colonies

Five-to-six-day-old mosquitoes, which had been allowed to mate in the cages, were starved at least 16 h as described above and then fed a meal of BB containing citrate dextrose solution (15:85 ratio to blood) as an anticoagulant (Lampire Biological Laboratories, Inc., Pipersville, PA) or a meal of SS. The mosquitoes were offered the meal for 1 h through a water-jacketed artificial membrane feeding system [43, 44] fitted with Parafilm M (Bernis Co. Inc. Nennah, WI) on 40-mm-diameter glass feeders (#1588–40, NDS Technologies, Vineland, NJ) and connected to a 37 °C SAHARA S7-heated bath circulator (ThermoFisher Scientific, Waltham, MA). *Aedes aegypti* females lay their eggs on a damp substrate; therefore, to collect their eggs, a paper cup filled with approximately 200 ml of distilled water and lined with filter paper was placed in each 1-gallon mosquito container immediately after feeding. On the 5th day after feeding, the filter paper was removed from the cup and dried for 24 h inside the environmental chamber. On the next day, the dried *Ae. aegypti* eggs were vacuum-hatched for 30 min.

*Anopheles stephensi* females deposit their eggs directly into the water; therefore, to collect their eggs a paper cup filled with 200 ml of distilled water was placed in each cage immediately after feeding. *Anopheles* *stephensi* larvae were present inside the cup on the 6th day after feeding.

Larvae of both species were transferred to shallow plastic pans and maintained as described above. After the adults from the initial 200 eggs were used to establish each replicate, the population of subsequent generations was maintained at approximately 400 mosquitoes with a balanced proportion of females and males.

### Collection of *Ae*. *aegypti* and *An*. *stephensi* engorgement and hatch rates data

Groups of approximately 20–40 females were selectively aspirated after attraction to the side of each gallon container by the warmth of a hand on the outside surface, transferred to plastic pint containers, and offered a BB or SS meal via an artificial membrane feeding system for 30 min. After feeding, the mosquitoes were anesthetized by placing the container inside a −20 °C freezer for 1.5 min. The mosquitoes were placed in a petri dish on ice. Engorged (fed) females were counted to determine engorgement rate (calculated by dividing the number of engorged females by the total number of females offered the meal) and separated to assess the number of eggs to be laid by each individual fed female.

After counting, the engorged females were individually placed into 50-ml conical centrifuge tubes containing a filter paper placed over a water-soaked cotton wool ball in the bottom. Each tube was capped with containment netting, and a second cotton wool ball wetted with 10% Karo^®^ syrup was placed on top (changed daily). The individual *Ae. aegypti* females were kept in their separate egg collection tubes for 4 days. On day 5, the females and water-soaked cotton wool balls from the bottom were discarded, and each clutch of eggs was collected on a filter paper. The eggs of each clutch were counted, and the individual papers were returned to their respective tubes to air dry overnight. On day 6, each dried egg paper was placed at the bottom of its respective tube, and 25–30 ml of distilled water was added to ensure that the paper was submerged. The tubes were vacuum hatched for 30 min and observed for larvae development.

*Anopheles stephensi* mosquitoes lay their eggs in water; therefore, the engorged counted females were placed individually into a disposable paper pint container with an egg collection cup (filter paper funnel placed inside a 30-ml plastic medicine cup filled with 15 ml distilled water). Each pint container was closed with a mesh top and sealed with a cardboard ring. The individual *An. stephensi* females were kept in their separate containers for 3 days for egg laying. On day 4, the females were removed, and the eggs of each clutch were counted and rinsed into a paper cup filled with 100 ml distilled water, where they were allowed to hatch.

For both mosquito species, on the day of hatch, and every other day afterward, a sprinkle of finely powdered Tetramin^®^ Tropical Flakes fish food was added to each tube or cup to ensure the growth of larvae. The larvae of the two species were counted 5–6 days post hatch. Average number of larvae per hatched egg was calculated by dividing the number of larvae 5–6 days after hatch by the total number of counted eggs.

### *Plasmodium gallinaceum* infections of *Ae*. *aegypti* mosquitoes

All animal experimental procedures were performed under protocols approved by the National Institute of Allergy and Infectious Diseases (NIAID) Animal Care and Use Committee. Animals were purchased from NIH-approved sources and transported and housed according to Guide for the Care and Use of Laboratory Animals [45].

*Plasmodium gallinaceum* strain 8A [46, 47] was maintained by continuous passage in 4- to 5-week-old white leghorn chickens (*Gallus gallus*). Approximately 60–100 *Ae. aegypti* mosquitoes 3–8 days old were transferred from respective replicate colonies into a new 1-gallon mosquito container and starved for at least 1 h prior. The mosquitoes were then allowed to feed directly on a ketamine/aceproprazine-sedated *P*. *gallinaceum*-infected chicken (10–20% parasitemia) through a mesh screen for 20 min. Immediately after feeding, approximately 30 fully engorged females were transferred into a new 1-gallon mosquito container and provided a cotton wool ball soaked with 10% Karo^®^ dark corn syrup daily.

### Quantification of *P. gallinaceum* oocysts and sporozoites in *Ae*. *aegypti* mosquitoes

Seven to 8 days after infection, the female mosquitoes were transferred by a battery-powered aspirator (Clarke no. 13500) to a half-gallon mosquito container and placed inside a −20 °C freezer for 1.5 min. The cold-anesthetized females were drowned in 70% ethanol for 2 min and then washed with 1 × phosphate-buffered saline (1 × PBS; 10 mM PO_4_^3−^, 137 mM NaCl, 2.7 mM KCl, pH 7.4). With the aid of a stereomicroscope (Olympus 5Z61, Olympus America Inc., Center Valley, PA), the female midguts were dissected with tweezers into 1 × PBS and stained for 30 min with a solution of 0.1% mercurochrome in distilled water, and oocysts were counted at 200 × magnification (20 × objective, 10 × oculars).

Fourteen to 15 days after infection, the remaining *Ae. aegypti* females were −20 °C anesthesized and drowned in 70% ethanol for 2 min, and the salivary glands were removed and collected in 50 µl of 1 × PBS. The number of lobes collected per female was recorded and pooled for each replicate. The lobes were milled for 1 min with a plastic disposable pestle, and 10 µl of each sample was pipetted and counted using a disposable hemocytometer as recommended by the manufacturer (Incyto C-Chip hemocytometers, SKC, Inc., Covington, GA).

### *Plasmodium falciparum* cultures

Human O + erythrocytes depleted of white blood cells were obtained weekly from Grifols Bio Supplies Inc. (Memphis, TN). The erythrocytes were washed upon arrival with 0.2-µM filtered RPMI 1640 medium (containing 25 mM HEPES and 50 µg/ml hypoxanthine; KD Medical, Columbia, MD) and stored at 50% hematocrit in a 4 °C refrigerator for use within a week from processing. Asynchronous cultures of the *P. falciparum* NF54 line [48] were maintained at 10-ml volumes in T25 vented flasks (Corning Inc. Life Sciences, Oneonta, NY) at 5% hematocrit with complete RPMI medium [RPMI 1640 medium supplemented with 10 mg/l gentamicin, 0.23% sodium bicarbonate, and 10% O + pooled human serum from 20 donors (Grifols Bio Supplies Inc.)]. Cultures were incubated at 37 °C under a 90% N_2_, 5% O_2_, and 5% CO_2_ gas mixture. Medium was changed daily. Parasitemias were monitored by methanol-fixed thin blood films stained for 15 min with 20% Giemsa solution (Sigma-Aldrich, St. Louis, MO) and maintained between 0.5 and 9% parasitemia.

### Induction of *P. falciparum* gametocytes

*Plasmodium falciparum* gametocytes were generated by “crash” induction in vitro [49, 50]. For this purpose, cultures of *P. falciparum* NF54 parasites were initiated as mixed stages in T75 flasks at 0.5% parasitemia and 5% hematocrit in the complete RPMI medium described above. Cultures were maintained with daily medium changes at 37 °C under a 90% N_2_, 5% O_2_, 5% CO_2_ gas mixture and monitored by methanol-fixed, Giemsa-stained thin blood films. Media changes were done on top of a slide warmer unit at 37 °C. When the stage V gametocytemia was prevalent at > 0.5% (days 14–16), the culture was collected for mosquito feeding.

### Infection of *An*. *stephensi* mosquitoes by *P. falciparum* gametocytes

All operations with infected live *An. stephensi* were performed inside a secure, triple-screened insectary. Approximately 20–40 uninfected *An. stephensi* females were transferred from each replicate colony maintained on BB or SS to a secure mosquito pint container (using a double mesh top secured with a metal ring) and starved for 16 h as described above. In some instances, when sufficient numbers of gametocytes and mosquitoes were available, additional pints of the same generation were prepared to increase the numbers of infected mosquitoes for evaluation (e.g. three pints each of BB- and SS-maintained *An. stephensi* at generation F8). The *P. falciparum*-infected blood meal was prepared as a 500-µl mixture containing one part heat-inactivated (56 °C × 30 min) O^+^ pooled human serum at 37 °C and one part of NF54 gametocytes in culture at 37 °C so that a final stage V gametocytemia of 0.1–0.3% was achieved. From this mixture, 250 µl was pipetted into a parafilm-sealed glass feeder at 37 °C as described above and offered to the 16 h-starved *An. stephensi* for 30 min. Counts of the engorged females were visually estimated, but, for safety, the pint containers were not opened to sort the engorged from non-engorged mosquitoes. After feeding, the pint containers of mosquitoes were placed inside a clear plastic bin (secondary containment) and stored inside the secure insectary. The mosquitoes were provided a 10% Karo^™^ dark corn syrup-soaked cotton wool ball daily.

### Quantification of *P. falciparum* oocysts and sporozoites in *An*. *stephensi* mosquitoes

Midgut oocysts of the infected *An*. *stephensi* were counted 6–8 days post infection, and salivary gland sporozoite assessments were performed 15–22 days post infection. For midgut dissections, the female mosquitoes were transferred via the battery-powered aspirator from each secure pint to a separate container and exposed to chloroform vapor for 1 min in a fume hood. The chloroform-anesthetized females were drowned in 70% ethanol for 1 min and then washed with 1 × PBS. Oocysts were stained and counted as described above. For sporozoite counts, salivary glands were extracted, and the number of lobes collected per female was recorded and pooled for each replicate. The lobes were milled for 1 min with a plastic disposable pestle, and the sporozoites were counted as described above.

### Statistical analysis

Data from colony replicates were recorded along with calculated averages or geometric means in Microsoft Excel workbook spreadsheets (Microsoft 365 online version 2208). Statistical modeling analyses were performed in R (version 4.3.0) [51]. Engorgement rates and parasite infectivity by oocysts were evaluated using generalized linear mixed models with binomial and negative binomial families, respectively, using package lme4 [52]. Egg hatch rates were evaluated using a generalized linear model with a quasibinomial link. We tested for potential interactions between meal and generation number in every model, and if an interaction was not found to be statistically significant, it was deleted from the model. In all mixed models, we considered the experimental variations of a given mosquito colony replicate and each individual mosquito to be a random effect and generation and meal to be fixed effects. To test whether an effect of the BB or SS meal significantly varied across generations, a likelihood ratio test (LRT) was performed in linear mixed models, and a deviance test was performed in the quasibinomial model. Sporozoites per mosquito were calculated separately for each colony, and t-test (*Ae. aegypti*) or weighted linear regression (*An. stephensi*) on the log-transformed colony rates was used to calculate geometric mean ratios (SS over BB) and confidence intervals. Further details are provided in Additional File 2: Statistical Appendix.

## Results

### Comparative engorgement rates, egg hatch rates, and *P*. *gallinaceum* infectivity to *Ae*. *aegypti* mosquito colonies maintained on SkitoSnack or bovine blood

**Fig. 1:**
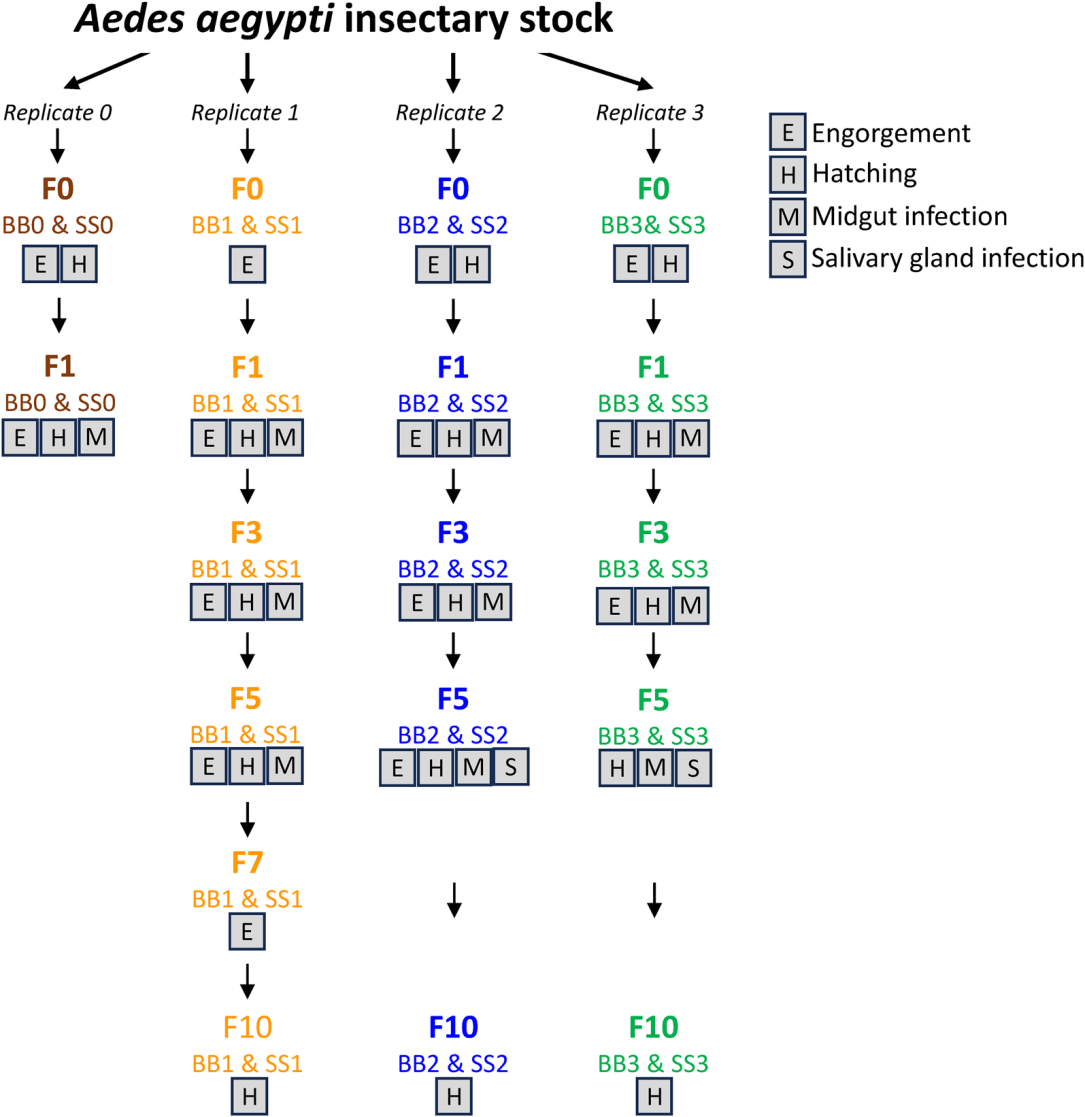
Flow diagram outlining the studies to assess the *Aedes aegypti* colony replicates maintained on SkitoSnack (SS) or bovine blood (BB). Labels indicate the generations for which mosquito meal engorgement rates, egg hatch rates, and *Plasmodium gallinaceum* oocyst counts and sporozoite counts were determined. The BB0 and SS0 replicates were lost at generation F2 because of a chamber thermoregulation failure in the insectary. Data were not collected from some replicates at other timepoints during periods of holidays, staff vacation and conference travel. Details of the results are listed in Additional file 3: Microsoft Excel workbook

Replicate colonies of *Ae. aegypti* were bred and maintained through multiple consecutive generations on either SS or BB (Fig. 1). Using these colonies, we assessed the comparative engorgement rates of female mosquitoes from one to four replicate colonies at generations F0 (immediately after the colonies were established), F1, F3, F5, and F7. The averages and IQR of these rates are presented in Table 1. By statistical analysis using a logistic regression mixed effects model, we found a significant effect due to meal, with a 2.6 (95% CI 1.3–5.2) greater odds of a mosquito engorging on the SS than the BB meal. The effect of the meal did not significantly vary across the generations (*p* = 0.89) according to likelihood ratio testing (Additional File 2: Statistical Appendix).

**Table 1:** Engorgement of *Aedes aegypti* from replicate colonies maintained on bovine blood (BB) or SkitoSnack (SS)^a^

Generation	Colony (# mosquitoes)	BB mosquitoes fraction engorged	Colony (# mosquitoes)	SS mosquitoes fraction engorged
F0	F0 BB0 (30)	0.77	F0 SS0 (35)	0.80
	F0 BB1 (32)	0.47	F0 SS1 (34)	0.50
	F0 BB2 (38)	0.45	F0 SS2 (35)	0.83
	F0 BB3 (18)	0.56	F0 SS3 (17)	0.76
		F0 BB avg (IQR): 0.56 (0.46–0.61)		F0 SS avg (IQR): 0.72 (0.70–0.81)
F1	F1 BB0 (26)	0.50	F1 SS0 (37)	0.73
	F1 BB1 (24)	0.21	F1 SS1 (21)	0.33
	F1 BB2 (17)	0.18	F1 SS2 (18)	0.67
	F1 BB3 (19)	0.68	F1 SS3 (20)	0.55
		F1 BB avg (IQR): 0.39 (0.20–0.55)		F1 SS avg (IQR): 0.57 (0.50–0.68)
F3	F3 BB1 (24)	0.88	F3 SS1 (30)	0.83
	F3 BB2 (19)	0.53	F3 SS2 (21)	0.62
	F3 BB3 (19)	0.26	F3 SS3 (18)	1.00
		F3 BB avg (IQR): 0.55 (0.39–0.70)		F3 SS avg (IQR): 0.82 (0.73–0.92)
F5	F5 BB1 (17)	0.88	F5 SS1 (17)	0.76
	F5 BB2 (19)	0.32	F5 SS2 (19)	0.95
		F5 BB avg (IQR): 0.60 (0.46–0.74)		F5 SS avg (IQR): 0.86 (0.81–0.90)
F7	F7 BB1 (16)	0.88	F7 SS1 (17)	0.88
		F7 BB1 only: 0.88		F7 SS1 only: 0.88

^a^Labels BB0–BB3 and SS0–SS3 identify individual replicate colonies; *avg*, average; *IQR*, interquartile range

Hatch rates data were collected from the SS- and BB-maintained *Ae. aegypti* colonies for assessments of offspring viability. The average egg hatch rates (IQR) at the F0, F1, F3, F5, and F10 generations are presented in Table 2. In statistical analysis using a quasibinomial model, a significantly lower odds of hatching was associated with the SS meal compared to BB (OR = 0.40, 95% CI 0.26–0.62). This effect of meal did not significantly vary across generations (*p* = 0.24) (Additional File 2: Statistical Appendix).

**Table 2:** Hatch rates of egg clutches from individual *Aedes aegypti* mosquitoes maintained on bovine blood (BB) or SkitoSnack (SS)^a^

Generation	Colony (# clutches)	BB hatch rate avg (IQR)	Colony (# clutches)	SS hatch rate avg (IQR)
F0	F0 BB0 (6)	0.90 (0.87–0.93)	F0 SS0 (6)	0.31 (0.23–0.31)
	F0 BB2 (4)	0.75 (0.72–0.83)	F0 SS2 (4)	0.73 (0.63–0.83)
	F0 BB3 (4)	0.79 (0.73–0.87)	F0 SS3 (5)	0.54 (0.51–0.69)
	combined F0 BB (14)	0.82 (0.79–0.89)	combined F0 SS (15)	0.50 (0.28–0.69)
F1	F1 BB0 (4)	0.85 (0.82–0.96)	F1 SS0 (6)	0.74 (0.66–0.86)
	F1 BB1 (4)	0.81 (0.72–0.90)	F1 SS1 (4)	0.91 (0.87–0.95)
	F1 BB2 (2)	0.57 (0.42–0.71)	F1 SS2 (5)	0.65 (0.54–0.75)
	F1 BB3 (8)	0.74 (0.70–0.82)	F1 SS3 (6)	0.63 (0.51–0.72)
	combined F1 BB (18)	0.76 (0.70–0.90)	combined F1 SS (21)	0.72 (0.60–0.86)
F3	F3 BB1 (5)	0.68 (0.57–0.80)	F3 SS1 (4)	0.40 (0.20–0.60)
	F3 BB2 (7)	0.66 (0.59–0.70)	F3 SS2 (8)	0.41 (0.29–0.51)
	F3 BB3 (1)	0.79 (—)	F3 SS3 (5)	0.83 (0.86–0.90)
	combined F3 BB (13)	0.68 (0.58–0.79)	combined F3 SS (17)	0.53 (0.36–0.64)
F5	F5 BB1 (7)	0.57 (0.31–0.84)	F5 SS1 (6)	0.50 (0.41–0.62)
	F5 BB2 (5)	0.81 (0.82–0.88)	F5 SS2 (7)	0.52 (0.37–0.59)
	F5 BB3 (9)	0.42 (0.24–0.65)	F5 SS3 (9)	0.45 (0.08–0.76)
	combined F5 BB (21)	0.56 (0.28–0.86)	combined F5 SS (22)	0.48 (0.36–0.64)
F10	F10 BB1 (11)	0.84 (0.82–0.93)	F10 SS1 (9)	0.74 (0.68–0.86)
	F10 BB2 (12)	0.84 (0.84–0.93)	F10 SS2 (10)	0.65 (0.50–0.78)
	F10 BB3 (8)	0.63 (0.31–0.90)	F10 SS3 (3)	0.53 (0.48–0.58)
	combined F10 BB (31)	0.78 (0.77–0.93)	combined F10 SS (22)	0.67 (0.55–0.81)

^a^Labels BB0–BB3 and SS0–SS3 identify individual replicate colonies; *avg*, Average; *IQR*, interquartile range

**Fig. 2:**
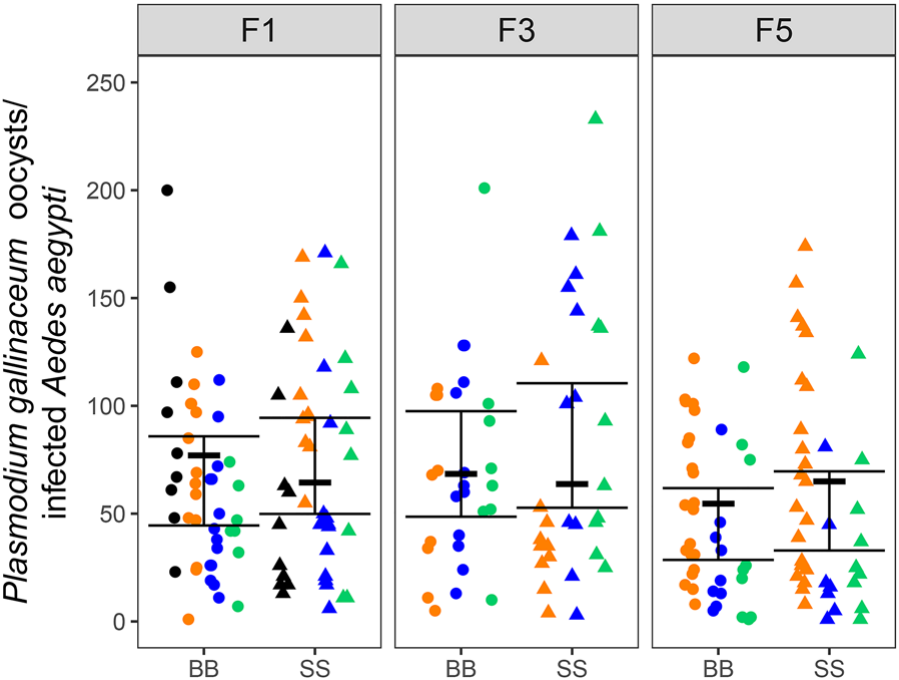
*Plasmodium gallinaceum* oocyst counts from replicate colonies of *Aedes aegypti* mosquitoes maintained on bovine blood (BB) or SkitoSnack (SS). Observed (dots) and statistically estimated (thick horizontal line) oocyst counts with bootstrap 95% confidence intervals (vertical lines) are shown for mosquito generations F1, F3, and F5. Colors represent the data from the different replicate populations fed on BB (circles) or SS (triangles)

Oocyst and sporozoite counts were obtained from replicate experiments in which *Ae. aegypti* mosquitoes from individual SS- and BB-maintained colonies were fed in parallel on a *P. gallinaceum*-infected chicken. Table 3 presents both the percentages of mosquitoes that became infected and the average (IQR) oocyst counts in the infected mosquitoes from the replicate colonies. For the BB- vs. SS-maintained *Ae. aegypti*, the oocyst-positive percentages were 100% vs. 98%, 100% vs. 100%, and 95% vs. 98% at generations F1, F3, and F5, respectively, and were not significantly different (all *p* > 0.6, see Additional File 2: Statistical Appendix). In a negative binomial model limited to mosquitoes with oocysts, the modeled ratio of mean oocyst counts per infected mosquito was not significantly different from 1 (mean ratio 1.11 × higher on SS compared to BB, 95% CI 0.85–1.49; *p* = 0.45) and the model found no evidence of variability in the effect of meal across generations (*p* = 0.99) (Additional File 2: Statistical Appendix). Figure 2 presents a summary display of the *P. gallinaceum* oocyst counts and statistical findings from the F1, F3, and F5 generations of the BB- and SS-maintained *Ae. aegypti*.

**Table 3:** Oocyst counts in *Plasmodium gallinaceum*-infected *Aedes aegypti* maintained on bovine blood (BB) or SkitoSnack (SS)^a^

Generation	Colony (number of mosquitoes dissected)	Fraction (%) with oocysts	BB avg oocysts per infected midgut (IQR)	Colony (number of mosquitoes dissected)	Fraction (%) with oocysts	SS avg oocysts per infected midgut (IQR)
F1	F1 BB0 (9)	9/9 (100%)	93 (61–111)	F1 SS0 (10)	10/10 (100%)	50 (18–62)
	F1 BB1 (14)	14/14 (100%)	68 (47–100)	F1 SS1 (11)	11/11 (100%	109 (89–137)
	F1 BB2 (14)	14/14 (100%)	48 (26–66)	F1 SS2 (13)	13/13 (100%)	55 (21–50)
	F1 BB3 (7)	7/7 (100%)	44 (37–55)	F1 SS3 (9)	8/9 (89%)	78 (34–112)
	combined F1 BB (44)	44/44 (100%)	63 (34–88)	combined F1 SS (43)	42/43 (98%)	72 (28–105)
F3	F3 BB1 (9)	9/9 (100%)	60 (34–105)	F3 SS1 (10)	10/10 (100%)	40 (28–44)
	F3 BB2 (12)	12/12 (100%)	70 (39–107)	F3 SS2 (10)	10/10 (100%)	96 (45–152)
	F3 BB3 (8)	8/8 (100%)	80 (52–95)	F3 SS3 (10)	10/10 (100%)	99 (47–137)
	combined F3 BB (29)	29/29 (100%)	70 (37–105)	combined F3 SS (30)	30/30 (100%)	79 (32–132)
F5	F5 BB1 (21)	21/21 (100%)	58 (31–85)	F5 SS1 (23)	23/23 (100%)	71 (25–111)
	F5 BB2 (10)	9/10 (90%)	29 (13–59)	F5 SS2 (7)	7/7 (100%)	26 (9–32)
	F5 BB3 (10)	9/10 (90%)	39 (2–75)	F5 SS3 (10)	9/10 (90%)	40 (18–52)
	combined F5 BB (41)	39/41 (95%)	47 (18–79)	combined F5 SS (40)	39/40 (98%)	56 (18–81)

^a^Labels BB0–BB3 and SS0–SS3 identify individual replicate colonies; *avg*, average; *IQR*, interquartile range

Sporozoite counts were obtained from small samplings from two colony replicates of the SS- and BB-maintained *Ae*. *aegypti* mosquitoes at generation F5 (Table 4). In our statistical analysis, the ratio of the geometric mean (GM) sporozoite count per SS-maintained mosquito divided by the GM sporozoite count per BB-maintained mosquito was 1.33 ×, but this ratio was not significantly different from 1 (GMR = 1.33, 95% CI 0.37–4.82) (Additional File 2: Statistical Appendix).

**Table 4:** *Plasmodium gallinaceum* sporozoite counts from dissected *Aedes aegypti* maintained on bovine blood (BB) or SkitoSnack (SS)^**a**^

Colony (# dissected mosquitoes)	Sporozoites/mosquito	Colony (# dissected mosquitoes)	Sporozoites/mosquito
F5 BB2 (2)	67.8 × 10^3^	F5 SS2 (2)	59.0 × 10^3^
F5 BB3 (3)	44.7 × 10^3^	F5 SS3 (3)	90.7 × 10^3^
	GM: 55.0 × 10^3^		GM: 73.1 × 10^3^

^a^Labels BB2–BB3 and SS2–SS3 identify individual replicate colonies; *GM* geometric mean

^b^Sporozoites were counted from ≤ 3 mosquitoes because staff were not available with the expertise to complete additional assessments

### Comparative engorgement rates, egg hatch rates, and *P. falciparum* infectivities to *An. stephensi* mosquito colonies maintained on SkitoSnack or bovine blood

**Fig. 3:**
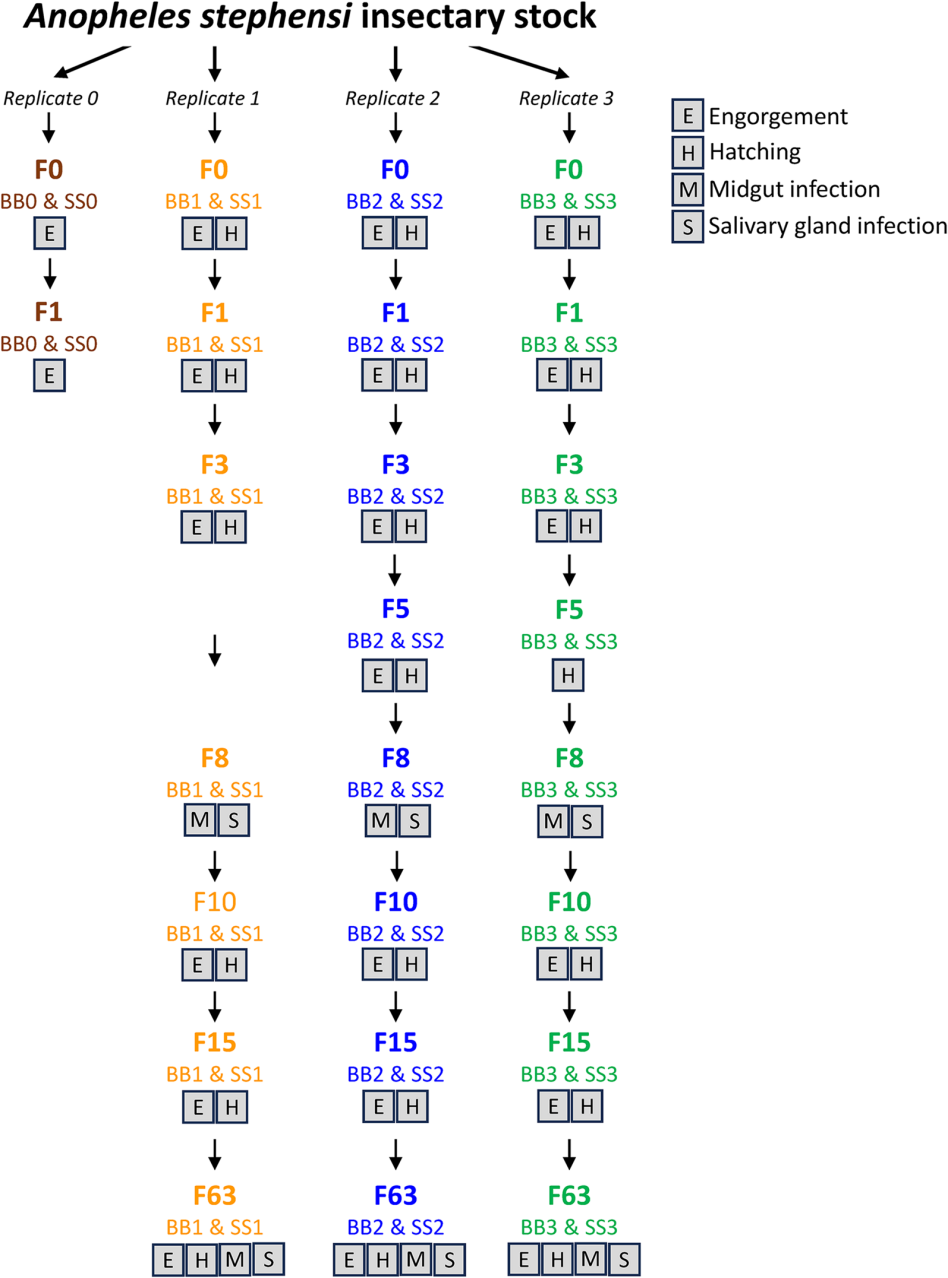
Flow diagram outlining the studies to assess the *Anopheles stephensi* colony replicates maintained on SkitoSnack (SS) or bovine blood (BB). Labels indicate the generations for which mosquito meal engorgement rates, egg hatch rates, and *Plasmodium falciparum* oocyst counts and sporozoite counts were determined. The BB0 and SS0 replicates were lost at generation F2 because of a chamber thermoregulation failure in the insectary. Data were not collected from some replicates at other timepoints during periods of holidays, staff vacation, and conference travel. Details of the results are listed in Additional file 3: Microsoft Excel workbook

**Fig. 4:**
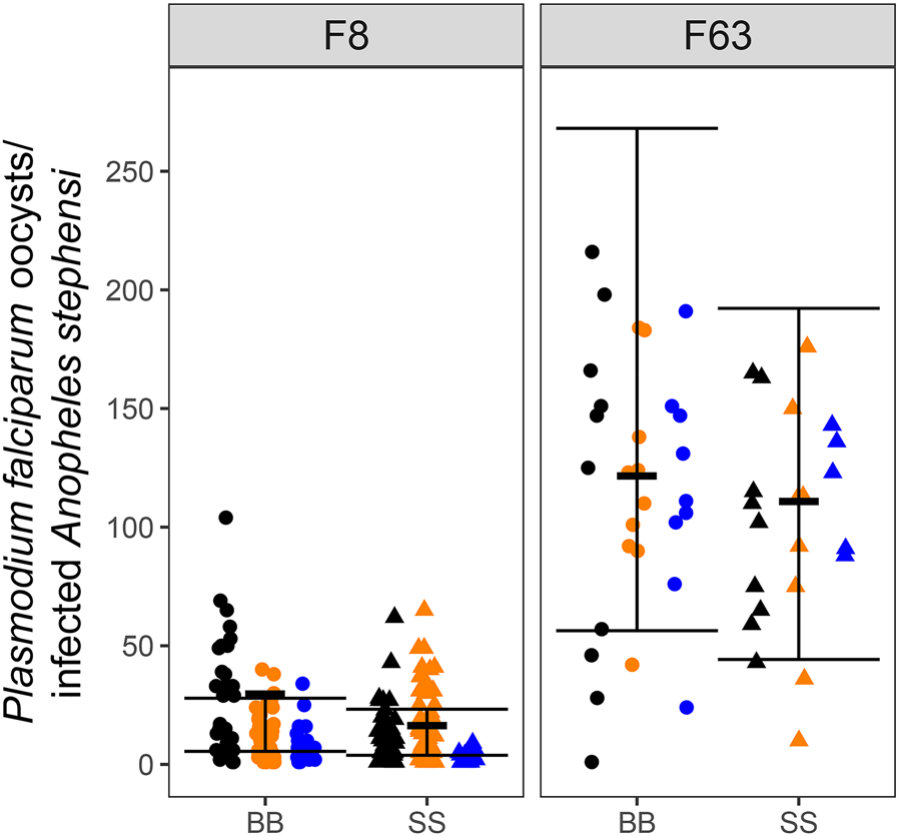
*Plasmodium falciparum* oocyst counts from replicate colonies of *Anopheles stephensi* mosquitoes maintained on bovine blood (BB) or SkitoSnack (SS). Observed (dots) and statistically estimated (thick horizontal line) oocyst counts with bootstrap 95% confidence intervals (vertical lines) are shown for mosquito generations F8 and F63. Colors represent the data from the different replicate populations fed on BB (circles) or SS (triangles)

Replicate colonies of *An. stephensi* were bred and maintained for > 63 generations on SS or BB (Fig. 3). Engorgement data were obtained from 1–4 replicate colonies at generations F0, F1, F3, F5, F10, F15, and F63; the averages and IQR of these rates are presented in Table 5. In the logistic regression mixed effects model, the LRT suggested that an effect of the meal varied across the generations (*p* = 0.02); therefore, comparisons between BB and SS were performed at each generation (Additional File 2: Statistical Appendix, Fig. 4). Although the interaction effect was significant, a meaningful pattern of the generational effects was not apparent. Because there were possibly random changes in the blood across generations (e.g. ATP levels), an overall averaging estimate of the meal effect was obtained by treating the interaction as part of the error. The odds of an *An. stephensi* mosquito engorging on SS were significantly greater than those of engorging on BB (OR = 2.71, 95% CI 1.4–5.51).

**Table 5:** Engorgement of *Anophelesi stephensi* from replicate colonies maintained on bovine blood (BB) or SkitoSnack (SS)^a^

Generation	Colony (# mosquitoes)	BB mosquitoes fraction engorged	Colony (# mosquitoes)	SS mosquitoes fraction engorged
F0	F0 BB0 (26)	0.73	F0 SS0 (33)	0.82
	F0 BB1 (35)	0.83	F0 SS1 (37)	1.00
	F0 BB2 (22)	0.55	F0 SS2 (31)	0.94
	F0 BB3 (17)	0.76	F0 SS3 (21)	0.90
		F0 BB avg (IQR): 0.72 (0.68–0.78)		F0 SS avg (IQR): 0.91 (0.88–0.95)
F1	F1 BB0 (31)	0.84	F1 SS0 (43)	0.79
	F1 BB1 (23)	0.48	F1 SS1 (25)	0.96
	F1 BB2 (22)	0.55	F1 SS2 (18)	1.00
	F1 BB3 (24)	0.92	F1 SS3 (29)	0.76
		F1 BB avg (IQR): 0.69 (0.53–0.86)		F1 SS avg (IQR): 0.88 (0.78–0.97)
F3	F3 BB1 (26)	0.58	F3 SS1 (26)	0.92
	F3 BB2 (31)	0.87	F3 SS2 (23)	1.00
	F3 BB3 (19)	0.84	F3 SS3 (22)	0.95
		F3 BB avg (IQR): 0.76 (0.71–0.86)		F3 SS avg (IQR): 0.96 (0.94–0.98)
F5	F5 BB2 (19)	0.89	F5 SS2 (28)	0.96
		F5 BB2 only: 0.89		F5 SS2 only: 0.96
F10	F10 BB1 (24)	0.75	F10 SS1 (33)	0.94
	F10 BB2 (20)	0.80	F10 SS2 (20)	1.00
	F10 BB3 (14)	0.71	F10 SS3 (25)	0.96
		F10 BB avg (IQR): 0.75 (0.73–0.78)		F10 SS avg (IQR): 0.97 (0.95–0.98)
F15	F15 BB1 (28)	0.86	F15 SS1 (24)	0.79
	F15 BB2 (30)	1.00	F15 SS2 (27)	1.00
	F15 BB3 (24)	0.63	F15 SS3 (22)	0.86
		F15 BB avg (IQR): 0.83 (0.74–0.93)		F15 SS avg (IQR): 0.89 (0.83–0.93)
F63	F63 BB1 (42)	0.88	F63 SS1 (47)	0.85
	F63 BB2 (43)	1.00	F63 SS2 (45)	0.96
	F63 BB3 (43)	1.00	F63 SS3 (57)	0.79
		F63 BB avg (IQR): 0.96 (0.94–1.00)		F63 SS avg (IQR): 0.87 (0.82–0.90)

^a^Labels BB0–BB3 and SS0–SS3 identify individual replicate colonies; *avg*, average; *IQR*, interquartile range

Egg hatch rates were obtained from 2–3 replicate colonies of *An. stephensi* at generations F0, F1, F3, F5, F10, F15, and F63. The average hatch rates (IQR) of these eggs at each generation are presented in Table 6. Using the quasibinomial model, analysis of the replicate datasets showed a significantly lower odds of hatching when the meal was SS compared to BB (OR = 0.59, 95% CI 0.36–0.96) and no evidence this effect varied across generations (*p* = 0.58) (Additional File 2: Statistical Appendix).

**Table 6:** Hatch rates of egg clutches from individual *Anopheles stephensi* mosquitoes maintained on bovine blood (BB) or SkitoSnack (SS)^a^

Generation	Colony (# clutches)	BB hatch rate avg (IQR)	Colony (# clutches)	SS hatch rate avg (IQR)
F0	F0 BB1 (7)	0.95 (0.95–0.96)	F0 SS1 (6)	0.83 (0.75–0.92)
	F0 BB2 (5)	0.78 (0.71–0.82)	F0 SS2 (5)	0.83 (0.78–0.92)
	F0 BB3 (4)	0.92 (0.91–0.99)	F0 SS3 (4)	0.84 (0.77–0.91)
	combined F0 BB (16)	0.89 (0.81–0.96)	combined F0 SS (15)	0.83 (0.77–0.92)
F1	F1 BB1 (5)	0.89 (0.83–0.93)	F1 SS1 (6)	0.85 (0.75–0.94)
	F1 BB2 (4)	0.93 (0.92–0.94)	F1 SS2 (6)	0.67 (0.55–0.87)
	F1 BB3 (2)	0.86 (0.86–0.87)	F1 SS3 (3)	0.61 (0.49–0.75)
	combined F1 BB (11)	0.90 (0.86–0.93)	combined F1 SS (15)	0.73 (0.61–0.92)
F3	F3 BB1 (7)	0.68 (0.63–0.70)	F3 SS1 (2)	0.72 (0.66–0.79)
	F3 BB2 (4)	0.83 (0.80–0.94)	F3 SS2 (4)	0.55 (0.31–0.83)
	F3 BB3 (5)	0.13 (0.00–0.23)	F3 SS3 (3)	0.03 (0.01–0.04)
	combined F3 BB (16)	0.55 (0.33–0.76)	combined F3 SS (9)	0.41 (0.07–0.80)
F5	F5 BB2 (4)	0.95 (0.94–0.97)	F5 SS2 (4)	0.96 (0.95–0.97)
	F5 BB3 (5)	0.90 (0.87–0.95)	F5 SS3 (6)	0.61 (0.45–0.83)
	combined F5 BB (9)	0.92 (0.88–0.97)	combined F5 SS (10)	0.75 (0.59–0.95)
F10	F10 BB1 (10)	0.71 (0.61–0.90)	F10 SS1 (12)	0.65 (0.52–0.77)
	F10 BB2 (4)	0.43 (0.35–0.55)	F10 SS2 (3)	0.28 (0.21–0.32)
	F10 BB3 (3)	0.64 (0.53–0.70)	F10 SS3 (3)	0.39 (0.26–0.50)
	combined F10 BB (17)	0.63 (0.49–0.85)	combined F10 SS (18)	0.54 (0.34–0.70)
F15	F15 BB1 (12)	0.74 (0.75–0.92)	F15 SS1 (6)	0.77 (0.64–0.88)
	F15 BB2 (8)	0.73 (0.59–0.92)	F15 SS2 (4)	0.76 (0.70–0.81)
	F15 BB3 (6)	0.75 (0.56–0.92)	F15 SS3 (11)	0.80 (0.68–0.96)
	combined F15 BB (26)	0.74 (0.60–0.92)	combined F15 SS (21)	0.78 (0.68–0.89)
F63	F63 BB1 (3)	0.55 (0.50–0.60)	F63 SS1 (3)	0.70 (0.67–0.76)
	F63 BB2 (3)	0.94 (0.93–0.95)	F63 SS2 (3)	0.53 (0.46–0.57)
	F63 BB3 (3)	0.51 (0.50–0.52)	F63 SS3 (3)	0.79 (0.75–0.82)
	combined F63 BB (9)	0.66 (0.51–0.91)	combined F63 SS (9)	0.67 (0.60–0.77)

^a^Labels BB0–BB3 and SS0–SS3 identify individual replicate colonies; *avg*, average; *IQR*, interquartile range

Oocyst counts were obtained from BB- or SS-maintained *An. stephensi* infected with *P*. *falciparum* gametocytes. Table 7 shows both the percentages of mosquitoes that became infected and the average (IQR) oocyst counts in the infected mosquitoes from three replicate colonies at the F8 and F63 generations. For the BB- vs. SS-maintained *An. stephensi*, the oocyst positive percentages were 87% vs. 91% and 97% vs. 78% at generations F8 and F63, respectively, and were not significantly different (*p* > 0.2 for both, see Additional File 2: Statistical Appendix). Large differences between the average oocyst counts in the infected mosquitoes of the two generations can be explained by variation of the gametocyte culture infectivity at the time of each experiment. In our analysis using the negative binomial model on counts from mosquitoes with oocysts, the mean oocyst count from the SS group was 0.76 × the mean from the BB group, but this was not significantly different from 1 (95% CI 0.44–1.33; *p* = 0.35) (Additional File 2: Statistical Appendix). Figure 4 presents a summary display of the *P. falciparum* oocyst counts and statistical findings from the F8 and F63 generations of the BB- and SS-maintained *An*. *stephensi*.

**Table 7:** Oocyst counts in *Plasmodium falciparum*-infected *Anopheles stephensi* maintained on bovine blood (BB) or SkitoSnack (SS)^a^

Generation	Colony (number of mosquitoes dissected)	Fraction (%) with oocysts	BB avg oocysts per infected midgut (IQR)	Colony (number of mosquitoes dissected)	Fraction (%) with oocysts	SS avg oocysts per infected midgut (IQR)
F8	F8 BB1 (32)	30/32 (94%)	28 (7–47)	F8 SS1 (37)	37/37 (100%)	13 (5–18)
	F8 BB2 (64)	54/64 (84%)	11 (3–16)	F8 SS2 (59)	56/59 (95%)	16 (5–25)
	F8 BB3 (32)	27/32 (84%)	8 (4–9)	F8 SS3 (30)	22/30 (73%)	3 (1–4)
	combined F8 BB (128)	111/128 (87%)	15 (4–19)	combined F8 SS (126)	115/126 (91%)	13 (3–18)
F63	F63 BB1 (10)	10/10 (100%)	114 (49–162)	F63 SS1 (9)	9/9 (100%)	100 (65–115)
	F63 BB2 (11)	10/11 (91%)	119 (94–135)	F63 SS2 (10)	8/10 (80%)	96 (65–122)
	F63 BB3 (9)	9/9 (100%)	115 (102–147)	F63 SS3 (9)	5/9 (56%)	116 (91–136)
	combined F63 BB (30)	29/30 (97%)	116 (90–151)	combined F63 SS (28)	22/28 (78%)	102 (75–133)

^a^Labels BB1–BB3 and SS1–SS3 identify individual replicate colonies; *avg*, average; *IQR*, interquartile range

Sporozoite counts were also obtained from *P. falciparum* infections of BB- or SS-maintained *An. stephensi* at generations F8 and F63. Table 8 presents the results from the three replicate colonies in each case. We found that the modeled geometric means of sporozoites per mosquito were similar between the SS- vs. BB-maintained *An. stephensi* mosquitoes (GMR = (gmean on SS)/(gmean on BB) = 1.30, 95% CI 0.39–4.27; *p* = 0.68) (Additional File 2: Statistical Appendix). The overall reduced number of sporozoites in the F8 vs. F63 mosquitoes is consistent with the much lower oocyst counts for the F8 infections.

**Table 8:** Sporozoite counts from dissected *Plasmodium falciparum*-infected *Anopheles stephensi* maintained on bovine blood (BB) or SkitoSnack (SS)^a, b^

Colony (# dissected mosquitoes)	Sporozoites/mosquito	Colony (# dissected mosquitoes)	Sporozoites/mosquito
F8 BB1 (8)	30.1 × 10^3^	F8 SS1 (18)	5.7 × 10^3^
F8 BB2 (5)	0.5 × 10^3^	F8 SS2 (7)	7.5 × 10^3^
F8 BB3 (8)	4.5 × 10^3^	F8 SS3 (9)	2.9 × 10^3^
	GM: 4.1 × 10^3^		GM: 5.0 × 10^3^
F63 BB1 (3)	52.3 × 10^3^	F63 SS1 (3)	48.7 × 10^3^
F63 BB2 (3)	34.1 × 10^3^	F63 SS2 (3)	103.2 × 10^3^
F63 BB3 (3)	29.3 × 10^3^	F63 SS3 (3)	27.0 × 10^3^
	GM: 37.4 × 10^3^		GM: 51.4 × 10^3^

^a^Labels BB1–BB3 and SS1–SS3 identify individual replicate colonies; *GM*, geometric mean

^b^Sporozoites were counted from the lobes of only three mosquitoes per F63 replicate because of limited staff availability during a holiday period

## Discussion

In this study, multiple consecutive generations of both *Ae*. *aegypti* and *An*. *stephensi* were grown and propagated using the blood-free meal, SS, as a replacement for the blood normally required to produce healthy, viable eggs. The *Ae. aegypti* mosquitoes were maintained for 10 generations before discontinuation, whereas the *An. stephensi* mosquitoes were maintained for > 63 generations,^1^ suggesting that mosquito colonies can thrive on SS many years, perhaps indefinitely. For all generations of both species, the mosquitoes maintained on SS were as robust and competent for *Plasmodium* infection, assessed both by midgut oocyst and salivary gland sporozoite counts, as mosquitoes maintained on BB.

Our findings add to growing evidence for the promise and potential value of SS-maintained mosquitoes in fundamental and applied vector research. *Aedes* *aegypti* mosquitoes effectively support infections with dengue virus after maintenance on SS for multiple generations [37]. Here, we have broadened the use of SS to the long-term maintenance of *An. stephensi*, in addition to *Ae*. *aegypti*, and show that SS colonies of these two evolutionary-distant species remain susceptible to *P*. *falciparum* (*An*. *stephensi*) or *P*. *gallinaceum* (*Ae*. *aegypti*). The manipulation of meal elements that is possible with SS may now support novel investigations of factors important to the mosquito life cycle and pathogen susceptibility. In insectaries with suitable containment conditions, tight control of microbiota in mosquito populations also may be feasible with SS.

The engorgement rates of *Ae. aegypti* and *An. stephensi* in our experiments were higher overall on SS than on BB. In a recent study, an *Ae. aegypti* colony raised on SkitoSnack for over 30 generations preferred a bovine blood meal over SkitoSnack when offered both meals in preference assays [36]. A limitation of our study is that it did not include such preference assays. Engorgement rates in the BB generations also may have been subject to different levels of ATP or other nucleotide phagostimulants [27, 53] whereas the ATP of SS was constant at 3 mM ATP. ATP variations as well as changes in the microbiome, which can affect feeding behavior as well as immunity and pathogen susceptibility [38], remain to be investigated in SS-maintained relative to BB-maintained mosquitoes.

Storage and preservation of eggs from SS-maintained mosquitoes may be desirable under some circumstances. In preliminary experiments, we have found that the eggs of SS-maintained *Ae*. *aegypti* can be desiccated and hatched > 3 months later, as has been reported for blood-maintained *Ae*. *aegypti* [54]. In contrast to the eggs of *Ae. aegypti*, the eggs of blood-maintained *An*. *stephensi* do not survive after desiccation and must be cryopreserved for storage [55]. Experiments have yet to show whether freshly oviposited eggs from SS-maintained *An. stephensi* can be cryopreserved similarly to eggs from BB-maintained *An. stephensi*.

Efficient and reliable maintenance of *Aedes* and *Anopheles* mosquitoes on blood-free meals avoids the necessity to acquire, handle, and preserve large amounts of vertebrate blood under refrigeration. Possible introductions of unwanted chemical or biological agents from blood are also circumvented. At relatively modest cost and without the logistical and ethical issues of blood supplies, the components of SS can be combined in bulk and stored dry at room temperature on the shelf until desired amounts are prepared with distilled water for feeding. Although the populations numbers in our study were relatively small, our results suggest SS may also be useful for mass-rearing of mosquito populations. Further testing will be needed to establish the feasibility and practicability of such scale up.

## Conclusions

Both *Ae. aegypti* and *An. stephensi* can be reliably propagated using blood-free SS as a replacement for the blood meal normally required for egg production. Mosquitoes maintained on SS for multiple generations (> 10 for *Ae*. *aegypti*; > 63 for *An. stephensi*) remained as robust and competent for *Plasmodium* infection as mosquitoes maintained on BB. Use of SS alleviates the need to acquire and preserve blood for mosquito husbandry and may support new initiatives in fundamental and applied research, including novel manipulations of midgut microbiota and factors important to the mosquito life cycle and pathogen susceptibility.

### Supplementary Information

Supplementary material 1

Supplementary material 2

Supplementary material 3

Supplementary material 4

## Data Availability

All data are provided in the Tables and Supplementary Information files.

## Footnotes

1 At the time of this publication, the SkitoSnack-maintained *An. stephensi* colonies are at their 92nd generation and thriving without having received any blood meal.
