# Supplementary material for: Infectivity of Plasmodium parasites to Aedes aegypti and Anopheles stephensi mosquitoes maintained on blood-free meals of SkitoSnack
Source: Parasit Vectors. 2024 Jul 6;17:290. doi: 10.1186/s13071-024-06364-9 (PMC11227701; doi:10.1186/s13071-024-06364-9)
Supplement: Supplementary file 1 — Supplementary material 1 [file 13071_2024_6364_MOESM1_ESM.docx]

**Table S1** SkitoSnack Preparation Protocol

SkitoSnack powder ingredients; measure

| **Components of SkitoSnack** | **Source (Company, Catalog no.)** | **Final Conc.** | **Final Conc. (g/mL)** | **To make 100 mL, weigh out (g)** |
| --- | --- | --- | --- | --- |
| Bovine serum albumin | **Research Products international. Cat# A30075-2502.0**. Web: https://www.rpicorp.com/products/biochemicals/biochemical-reagents/albumin-bovine-fraction-v-250-g.html | 200 mg/ml | 0.2 | 20.000 |
| Bovine hemoglobin | **Sigma Aldrich**. **Cat# H3760**. Web: https://www.sigmaaldrich.com/US/en/product/sigma/h3760 | 5 mg/ml | 0.005 | 0.500 |
| Chicken yolk | **Sigma Aldrich. Cat# E0625**. Web: https://www.sigmaaldrich.com/US/en/product/sigma/e0625 | 5 mg/ml | 0.005 | 0.500 |
| Glucose | **Sigma Aldrich. Cat# G7021.** Web: https://www.sigmaaldrich.com/US/en/product/sigma/g7021 | 50.0 mM | 0.009008 | 0.901 |
| Adenosine triphosphate | **Sigma Aldrich. Cat# A6419.** Web: https://www.sigmaaldrich.com/US/en/product/sigma/a6419 | 3.0 mM | 0.0016534 | 0.165 |
| Sodium chloride | **Sigma Aldrich. Cat# S7653.** Web: https://www.sigmaaldrich.com/US/en/product/sial/s7653 | 150.0 mM | 0.008766 | 0.877 |
| Sodium bicarbonate | **Sigma Aldrich. Cat# S6297.** Web: https://www.sigmaaldrich.com/US/en/product/sial/s6297 | 23.0 mM | 0.0019322 | 0.193 |
| Potassium chloride | **Sigma Aldrich. Cat# P9333.** Web: https://www.sigmaaldrich.com/US/en/product/sial/p9333 | 4.0 mM | 0.0002982 | 0.030 |
| Calcium chloride | **Sigma Aldrich. Cat# C1016.** Web: https://www.sigmaaldrich.com/US/en/product/sigald/c1016 | 2.5 mM | 0.0002774 | 0.028 |
| Magnesium chloride | **Sigma Aldrich. Cat# M8266.** Web: https://www.sigmaaldrich.com/US/en/product/sigma/m8266 | 0.8 mM | 0.0000761 | 0.008 |
| **TOTAL** |  | **1x** | **0.232** | **23.200** |

Combine all ingredients and grind with mortar and pestle into a fine powder

(if grinding is skipped, ingredients will take longer to dissolve).

1. Weigh out 0.232 g of SkitoSnack powder for every 1 mL of meal (e.g. if you want to make 4 mL, use 0.93 g)
2. Put SkitoSnack powder into a round bottom tube.

*it is helpful to mark a heavy line at desired volume because original marks are hard to see on tube after SkitoSnack is reconstituted.

1. Add distilled water to the final volume line (e.g. 4 mL), then vortex until SkitoSnack powder is fully dissolved.
2. Add more distilled water if needed to reach final volume (e.g. 4 mL).
3. Pipet into artificial membrane feeder immediately.

*we consider dissolved SkitoSnack to have a bench life of 3 hours.
