# Supplementary material for: Infectivity of Plasmodium parasites to Aedes aegypti and Anopheles stephensi mosquitoes maintained on blood-free meals of SkitoSnack
Source: Parasit Vectors. 2024 Jul 6;17:290. doi: 10.1186/s13071-024-06364-9 (PMC11227701; doi:10.1186/s13071-024-06364-9)
Supplement: Supplementary file 3 — Supplementary material 3 [file 13071_2024_6364_MOESM3_ESM.pdf]

Infectivity of *Plasmodium* parasites to *Aedes aegypti* and *Anopheles stephensi* mosquitoes maintained on blood-free meals of SkitoSnack

Statistical Appendix

2024-04-30

1 Introduction

There are 4 main types of effects we are measuring (engorgement rates, hatch rates, oocyst counts per infected midgut, and sporozoites per infected mosquito). We list the data by generation and colony in the main tables of the paper (Tables 1-4). In those Tables we use averages and interquartile ranges throughout.

For hatch rates and oocyst counts, there are two levels of summarization. For example, for hatch rates we first take the average and interquartile range (IQR) of groups of eggs. These are presented for each generation/colony row. Then we take the unweighted average and IQR of those averages within each generation. Oocyst counts averages and IQRs are done similarly. We use IQR throughout Tables 1-4 for consistency, even though it is non-standard to calculate an IQR with only 2-4 observations.

For comparing the effects of the meal, bovine blood versus SkitoSnack, the analyses for each of the 4 types of effects are different and necessarily more sophisticated, so that each can properly account for any of the types of variability in its measurement (e.g., variation between colonies, generations, mosquitoes, eggs, oocysts, and sporozoites). We provide the details of those analyses in separate sections of this supplement. These analyses were performed in R version 4.3.0 (2023-04-21).

2 Engorgement Rate

2.1 Analysis Details

- In the sections below we present analyses to determine whether the type of meal (BB: bovine blood or SS: SkitoSnack) has an effect on engorgement rates for *Aedes aegypti* and *Anopheles stephensi*.
- We consider a logistic regression mixed effects model.
  - *Random effects*: In our work, considered to be factors (or categorical variables) with levels that are a random sample from some population. These are the colony replicates for each generation.
  - *Fixed effects*: In our work, considered to be factors with treatments that are pre-specified by the researcher and are constant across individuals (mosquitoes). These are meal and generation.
- The models were fit using the package lme4, citation: Bates D, Mächler M, Bolker B, Walker S (2015). "Fitting Linear Mixed-Effects Models Using lme4." *Journal of Statistical Software*, 67(1), 1–48.
- The 95% confidence intervals for the estimated probabilities were obtained via bootstrap with 1000 iterations.
- The reference meal is BB and reference generation is F0.
- A likelihood ratio test (LRT) was performed to assess an interaction between generation and meal.
- An LRT was performed to assess the overall generation effect.

2.2 *Aedes aegypti*

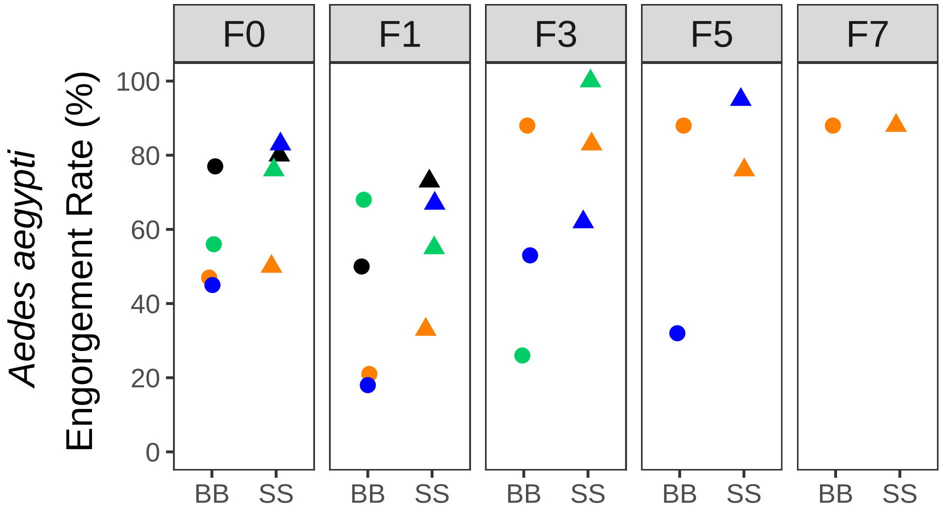

Figure 1: *Aedes aegypti* engorgement rate (%). Circles and triangles represent the data from the different replicate colonies fed on BB (circles), or SS (triangles), respectively. The colors correspond to the replicates which match the colors used in Additional file 3: Microsoft Excel workbook.

- We performed an LRT to determine whether there was a significant interaction between generation and meal. The p-value of the test is p: 0.895.
- We found a significant effect due to meal. The odds of a mosquito engorging when the meal is SS are 2.6 (95%CI:[1.33,5.23]) times higher than when the meal is BB.
- We found a significant effect due to generation from a LRT p: 0.034. This is an overall effect, though each individual test of each generation to F0 may not be significant as displayed on the table below.

Table 1: *Aedes aegypti* summary table in OR scale

	OR	Lower	Upper	p.value
SS vs. BB	2.605	1.328	5.227	0.004
F1 vs. F0	0.480	0.198	1.134	0.084
F3 vs. F0	1.385	0.535	3.715	0.491
F5 vs. F0	1.709	0.560	5.502	0.339
F7 vs. F0	4.294	0.886	23.653	0.073

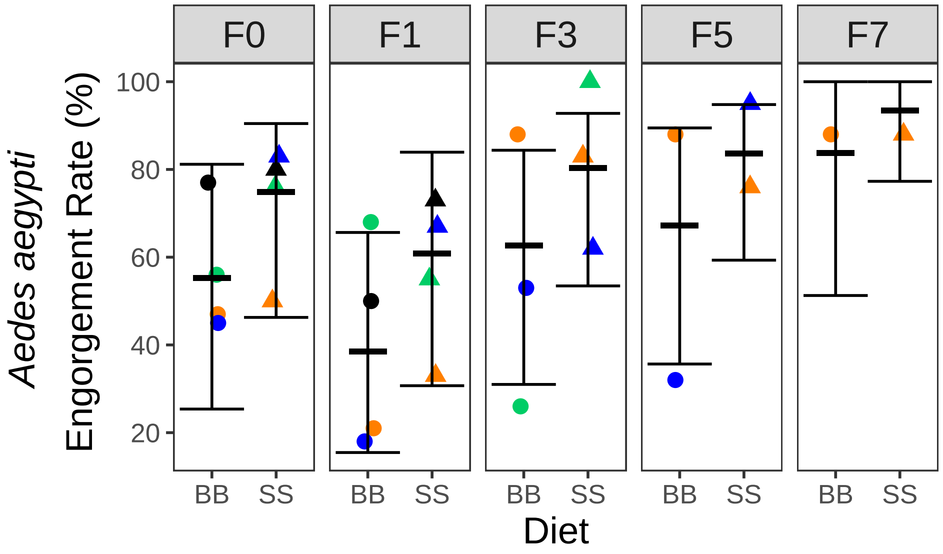

Figure 2: Observed (dots) and estimated (horizontal line) engorgement rates and bootstrap 95% confidence intervals (vertical lines) for *Aedes aegypti*. Circles and triangles represent the data from the different replicate colonies fed on BB (circles), or SS (triangles), respectively. The colors correspond to the replicates which match the colors used in Additional file 3: Microsoft Excel workbook.

2.3 *Anopheles stephensi*

- We performed an LRT to determine whether there was a significant interaction between generation and meal. The p-value of the test is p: 0.023.
- Because of our significant interaction results, we performed individual contrasts to test whether there is a significant difference between SS and BB at each generation.

Table 2: Engorgement odds ratios and 95% confidence intervals associated with SS vs BB at each generation for *Anopheles stephensi*

	OR	Lower	Upper	p.value
F0: SS vs BB	4.81	1.41	16.48	0.01
F1: SS vs BB	2.91	0.90	9.45	0.08
F3: SS vs BB	7.58	1.41	40.74	0.02
F5: SS vs BB	3.31	0.15	74.06	0.45
F10: SS vs BB	9.26	1.68	51.14	0.01
F15: SS vs BB	1.51	0.35	6.43	0.58
F63: SS vs BB	0.22	0.05	1.00	0.05

- Although the generation effect appears to be significant as shown in the LRT and the above figures, we do not see a meaningful pattern. Therefore, to get an overall averaging estimate of the meal effect we treat the interaction as part of the error.

Table 3: *Anopheles stephensi* summary table in OR scale

	OR	Lower	Upper	p.value
SS vs. BB	2.712	1.399	5.508	0.003
F1 vs. F0	0.785	0.275	2.272	0.640
F3 vs. F0	1.495	0.464	5.019	0.493
F5 vs. F0	2.923	0.461	21.996	0.261
F10 vs. F0	1.482	0.455	4.975	0.505
F15 vs. F0	1.419	0.447	4.693	0.545
F63 vs. F0	2.232	0.721	7.315	0.157

- We found a significant effect due to meal. The odds of a mosquito engorging when the meal is SS are 2.71 (95%CI:[1.4,5.51]) times higher than when the meal is SS.

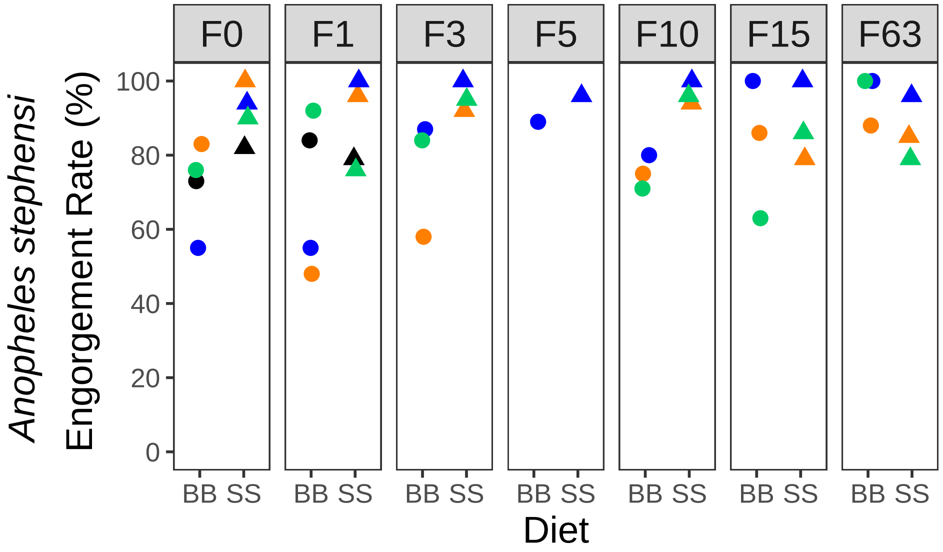

Figure 3: *Anopheles stephensi* engorgement rate (%). Circles and triangles represent the data from the different replicate colonies fed on BB (circles), or SS (triangles), respectively. The colors correspond to the replicates which match the colors used in Additional file 3: Microsoft Excel workbook.

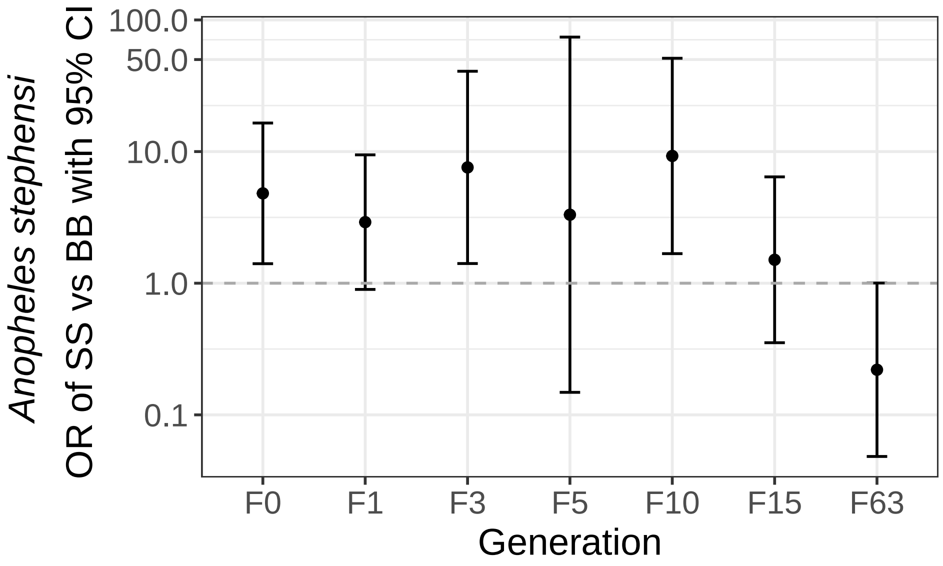

Figure 4: Engorgement odds ratios and 95% confidence intervals associated with SS vs BB at each generation for *Anopheles stephensi*

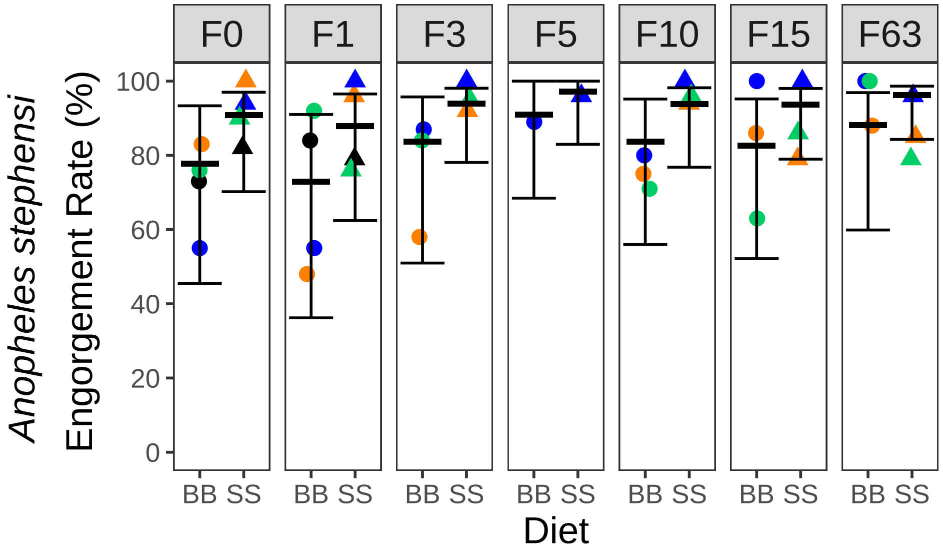

Figure 5: Observed (dots) and estimated (horizontal line) engorgement rates and bootstrap 95% confidence intervals (vertical lines) for *Anopheles stephensi*. Circles and triangles represent the data from the different replicate colonies fed on BB (circles), or SS (triangles), respectively. The colors correspond to the replicates which match the colors used in Additional file 3: Microsoft Excel workbook.

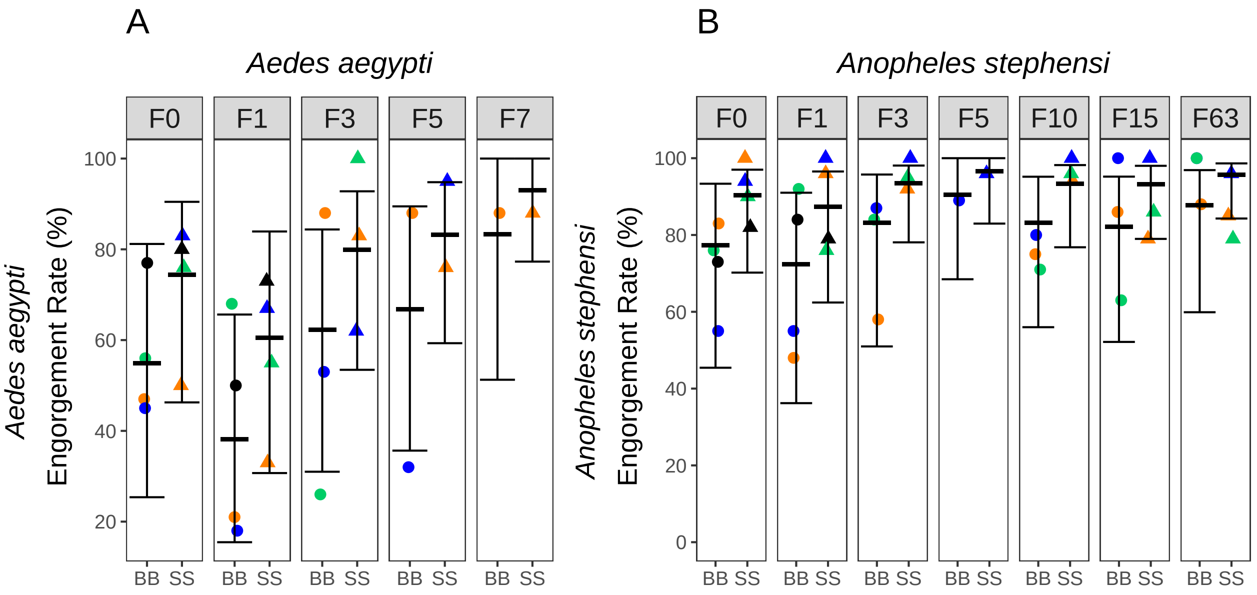

Figure 6: Engorgement rates of *Aedes aegypti* and *Anopheles stephensi* female mosquitoes offered meals of bovine blood (BB) or SkitoSnack (SS). A. Results from 1-4 biological replicate colonies of *Aedes aegypti* at generations F0, F1, F3, F5, and F7. B. Results from 1-4 biological replicate colonies of *Anopheles stephensi* at generations F0, F1, F3, F5, F10, F15, and F63. Colors represent the data from the different replicate colonies fed on BB (circles), or SS (triangles). Horizontal lines mark the estimated the estimated engorgement rate and vertical lines mark the bootstrap 95% confidence intervals of the engorgement rate.

3 Hatch Rate

3.1 Analysis Details

- In the sections below we present analyses to determine whether the type of meal (BB: bovine blood or SS: SkitoSnack) has an effect on hatch rate.
- We consider a logistic regression with quasibinomial family for our variable of interest.
- We include a generation by meal interaction to estimate the effect of diet at each generation and use a test of deviance to assess whether the interaction is significant.
- If the interaction is not significant it is removed from the model.
- Reference values: The reference for Meal is BB and for Generation is F0.

3.2 *Aedes aegypti*

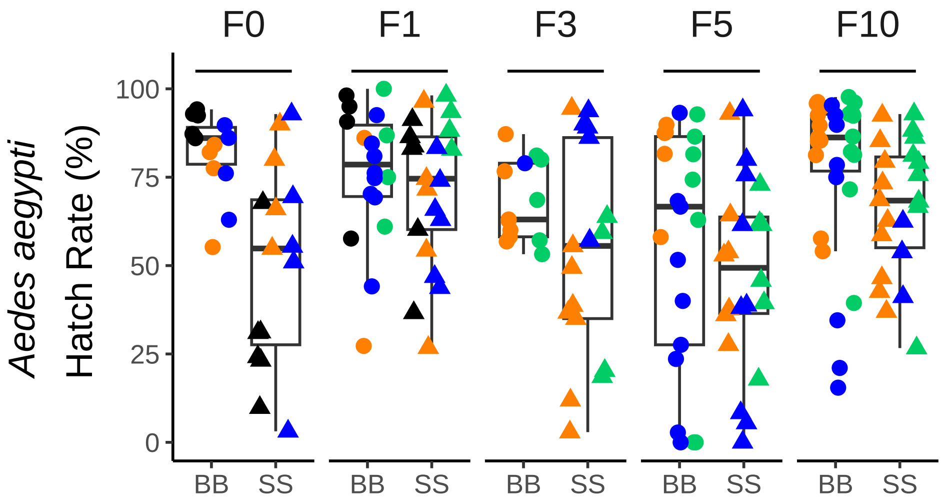

Figure 7: Observed hatch rates (%) for *Aedes aegypti*. Circles and triangles represent the data from the different replicate colonies fed on BB (circles), or SS (triangles), respectively. The colors correspond to the replicates which match the colors used in Additional file 3: Microsoft Excel workbook.

- We did not find sufficient evidence of a significant interaction, test of deviance p: 0.239.
- We found evidence of a significant generation effect, test of deviance p: 0.023.
- We found evidence of significant effect of meal p:<0.001. The odds of an egg hatching are 0.4 times lower (95%CI:[0.26,0.62]) when the meal is SkitoSnack when compared to bovine blood.

Table 4: *Aedes aegypti* hatch rates model summary

	OR	Lower	Upper	p.value
(Intercept)	3.04	1.82	5.24	<0.001
MealSS	0.40	0.26	0.62	<0.001
GenerationF1	1.65	0.81	3.43	0.181
GenerationF3	0.67	0.32	1.39	0.293
GenerationF5	0.62	0.32	1.18	0.161
GenerationF10	1.34	0.70	2.58	0.379

- Below we present observed hatch rates with estimated hatch rates and 95% bootstrap confidence intervals.

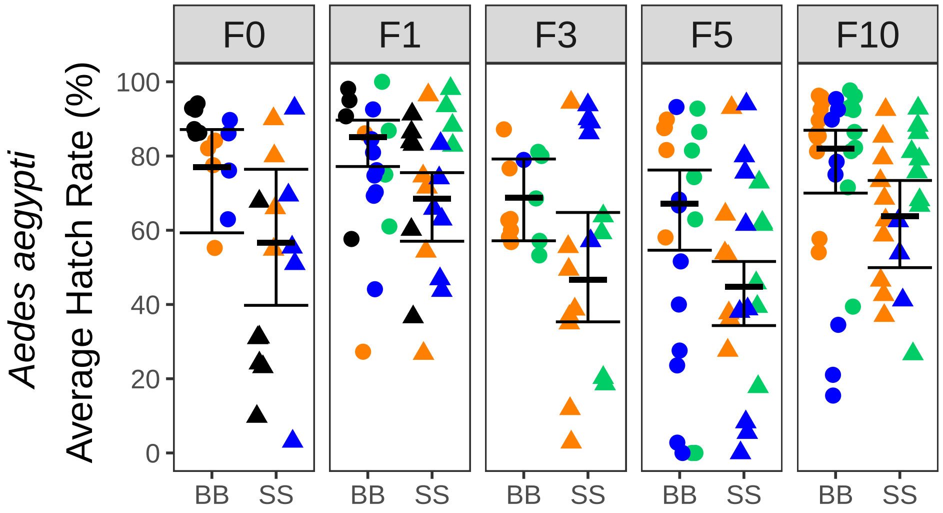

Figure 8: Hatch rates and 95% bootstrap confidence intervals for *Aedes aegypti*. Circles and triangles represent the data from the different replicate colonies fed on BB (circles), or SS (triangles), respectively. The colors correspond to the replicates which match the colors used in Additional file 3: Microsoft Excel workbook.

3.3 *Anopheles stephensi*

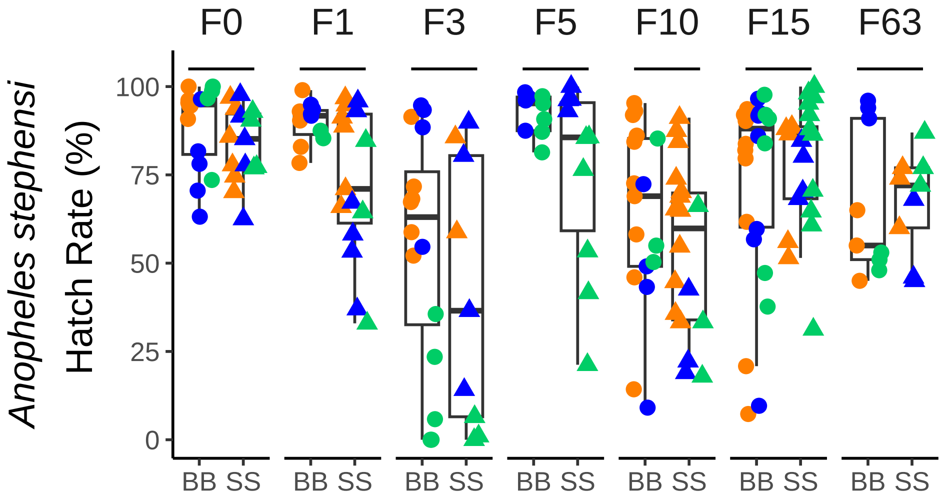

Figure 9: Observed hatch rates for *Anopheles stephensi*. Circles and triangles represent the data from the different replicate colonies fed on BB (circles), or SS (triangles), respectively. The colors correspond to the replicates which match the colors used in Additional file 3: Microsoft Excel workbook.

- We found no significant interaction. Test of deviance p: 0.581.
- We found evidence of a significant generation effect. Test of deviance p: <0.001.
- We found evidence of a significant effect of meal p:0.041. The odds of an egg hatching are 0.59 times lower (95%CI:[0.36,0.96]) when the meal is SkitoSnack when compared to bovine blood.

Table 5: *Anopheles stephensi* hatch rate model summary

	OR	Lower	Upper	p.value
(Intercept)	8.316	4.026	19.411	<0.001
MealSS	0.586	0.357	0.956	0.041

	OR	Lower	Upper	p.value
GenerationF1	0.600	0.216	1.622	0.323
GenerationF3	0.139	0.052	0.344	<0.001
GenerationF5	0.689	0.224	2.200	0.521
GenerationF10	0.227	0.089	0.535	0.003
GenerationF15	0.510	0.192	1.281	0.169
GenerationF63	0.322	0.112	0.886	0.037

- Below we present observed hatch rates with estimated hatch rates and 95% bootstrap confidence intervals.

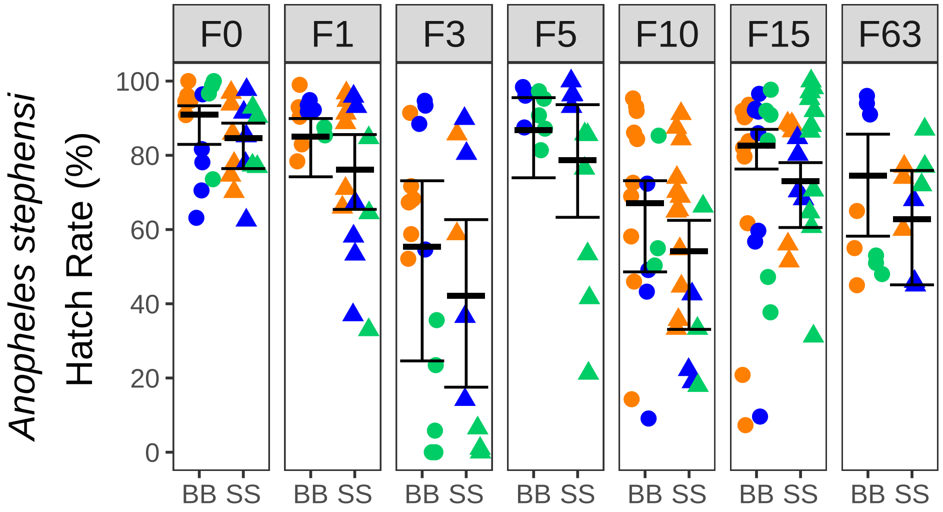

Figure 10: Observed (dots) and estimated (horizontal line) hatch rates and bootstrap 95% confidence intervals (vertical lines) for *Anopheles stephensi*. Circles and triangles represent the data from the different replicate colonies fed on BB (circles), or SS (triangles), respectively. The colors correspond to the replicates which match the colors used in Additional file 3: Microsoft Excel workbook.

3.4 Summary Figure

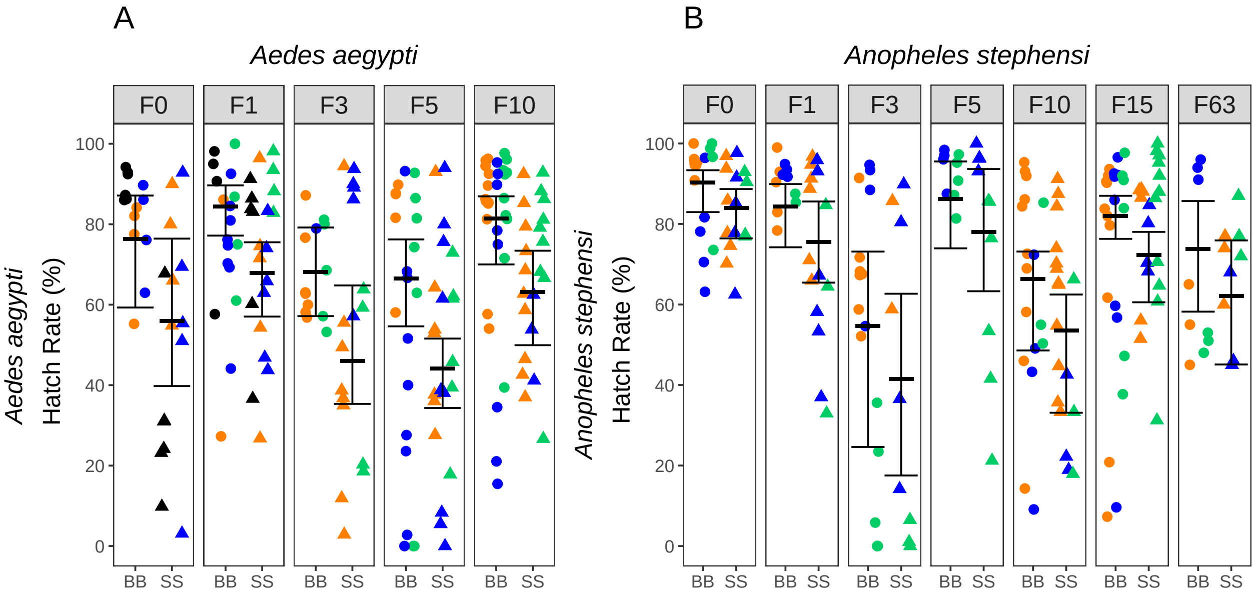

Figure 11: Hatch rates of *Aedes aegypti* and *Anopheles stephensi* female mosquitoes offered meals of bovine blood (BB) or SkitoSnack (SS). A. Results from 3-4 biological replicate colonies of *Aedes aegypti* at generations F0, F1, F3, F5, and F10. B. Results from 2-3 biological replicate colonies of *Anopheles stephensi* at generations F0, F1, F3, F5, F10, F15, and F63. Colors represent the data from the different replicate colonies fed on BB (circles), or SS (triangles). Horizontal lines mark the estimated hatch rates and vertical lines mark bootstrap 95% confidence intervals of the hatch rate.

4 Vector Susceptibility Analysis - Oocyst Counts

4.1 Analysis Details

- In the sections below we present analyses to determine whether the type of meal (BB: bovine blood or SS: SkitoSnack) has an effect on oocyst counts.
- We are using a negative binomial model, where our response is the count of oocysts in the midgut.
- We consider a mixed effects model to control for the correlation within groups or similar factors.
  - *Random effect*: Replicate colonies for each generation
  - *Fixed effect*: Meal and generation.
- The models were fit using the package lme4, citation: Bates D, Mächler M, Bolker B, Walker S (2015). "Fitting Linear Mixed-Effects Models Using lme4." *Journal of Statistical Software*, 67(1), 1–48.
- The 95% confidence intervals for the estimated probabilities were obtained via bootstrap with 1000 iterations.
- For all analyses the reference meal is bovine blood.
- The analysis is limited to infected mosquitoes, therefore mosquitoes with zero oocyst counts were eliminated.
- We performed a vector susceptibility sensitivity analysis to compare the fraction of dissected mosquitoes with oocysts present between the BB and SS meal groups using a quasi-binomial model within each mosquito species/generation combination.

4.2 *Aedes aegypti*

- The reference generation is F5

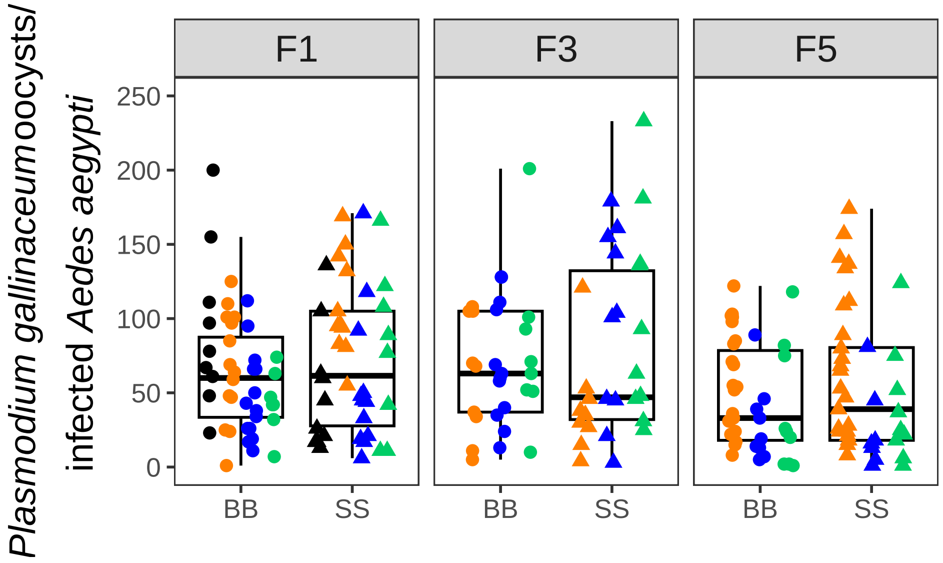

Figure 12: Observed oocyst counts for *Aedes aegypti*. Circles and triangles represent the data from the different replicate colonies fed on BB (circles), or SS (triangles), respectively. The colors correspond to the replicates which match the colors used in Additional file 3: Microsoft Excel workbook.

- We performed a likelihood ratio test (LRT) to determine whether there was a significant interaction between generation and meal. The p-value of the test is p: 0.989.
- Because of our interaction results we will work with a model that does not include an interaction term.

Table 6: Oocysts count estimates and 95% bootstrap confidence intervals for *Aedes aegypti*

	Estimate	Lower	Upper	P.value
(Intercept)	3.768	3.460	4.032	0.000
MealSS	0.108	-0.171	0.382	0.451
Generation2F1	0.366	0.038	0.685	0.036
Generation2F3	0.458	0.093	0.840	0.015

- We don't have sufficient evidence to say there is a significant difference between meals (p: 0.451).
- However, our best estimate of the meal effect is that changing the meal from BB to SS would increase the number of oocysts in the midgut on average by a factor of $e^{\beta_{SS}} \rightarrow 1.114$. This means that the mean number of oocysts in the midgut is 1.114 times higher when the meal is SS than when the meal is BB with 95% confidence bounds [0.84,1.448].
- There is a significant generation effect.
  - Generation F1 significantly differs from F5 p: 0.036. This means that the mean number of oocysts in the midgut is 1.442 times higher in generation F1 than in F5, the 95% CI is [1.038,1.983].
  - Generation F3 significantly differs from F5 p: 0.015. This means that the mean number of oocysts in the midgut is 1.581 times higher in generation F3 than in F5, the 95% CI is [1.098,2.315].
- The plot below presents the observed oocysts counts by generation, along with oocyst count estimates (dark horizontal line) and bootstrap 95% confidence intervals.

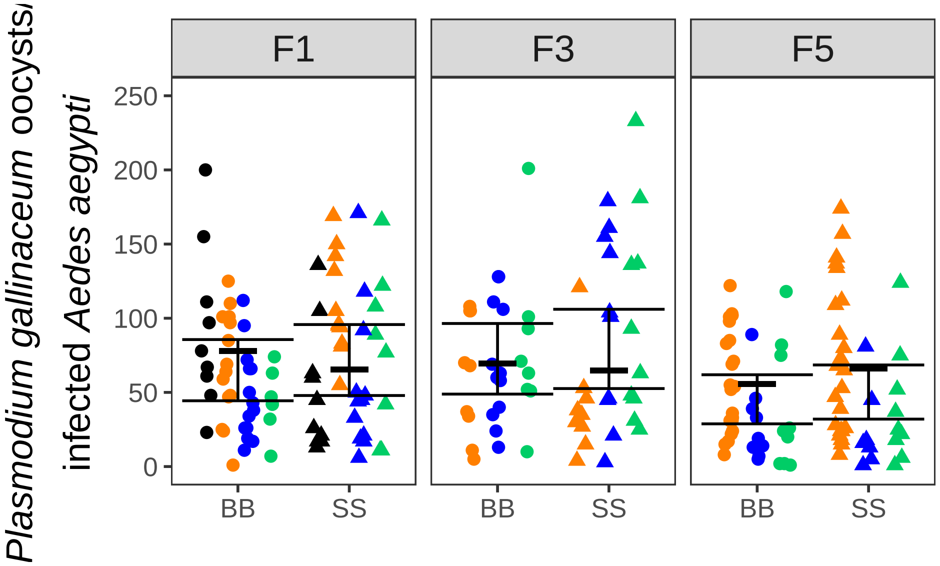

Figure 13: Observed (dots) and estimated (horizontal line) oocyst counts and bootstrap 95% confidence intervals (vertical lines) for *Aedes aegypti*. Circles and triangles represent the data from the different replicate colonies fed on BB (circles), or SS (triangles), respectively. The colors correspond to the replicates which match the colors used in Additional file 3: Microsoft Excel workbook.

4.2.1 Vector Susceptibility Sensitivity Analysis

Table 7: p-values from the quasi-binomial model comparing, for each generation, the proportion of dissected mosquitoes with oocysts from the two meal groups for *Aedes aegypti*

	p-value
Generation: F1	0.998
Generation: F3	1.000
Generation: F5	0.654

We found no significant differences in the fraction of infected mosquitoes at any of the three generations.

4.3 *Anopheles stephensi*

- The generation reference is F8.

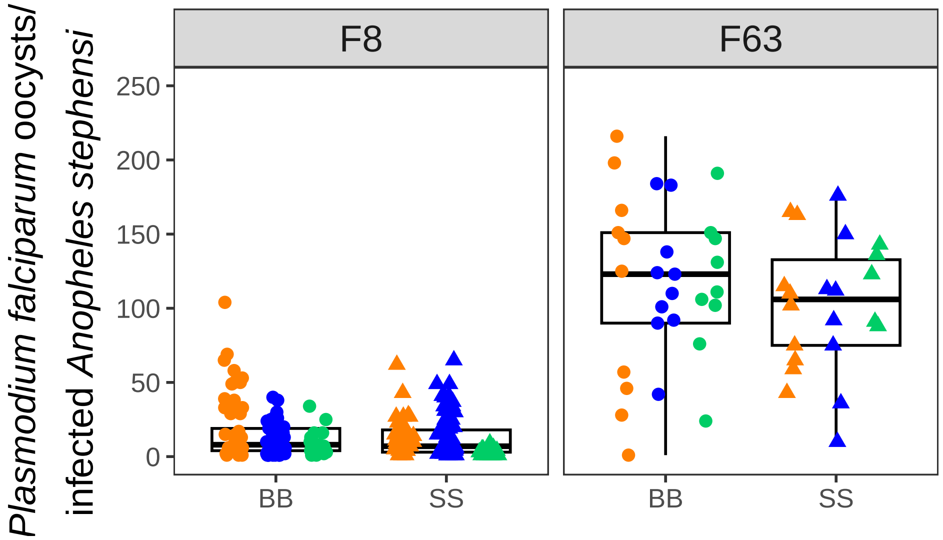

Figure 14: Observed oocyst counts for *Anopheles stephensi*. Circles and triangles represent the data from the different replicate colonies fed on BB (circles), or SS (triangles), respectively. The colors correspond to the replicates which match the colors used in Additional file 3: Microsoft Excel workbook.

- We performed a likelihood ratio test (LRT) to determine whether there was a significant interaction between generation and meal. The p-value of the test is p: 0.638.
- Because of our interaction results we will work with a model that does not include an interaction term.

Table 8: Oocysts count estimates and 95% bootstrap confidence intervals for *Anopheles stephensi*

	Estimate	Lower	Upper	P.value
(Intercept)	2.535	2.053	2.991	<0.001
MealSS	-0.273	-0.826	0.308	0.346
GenerationF63	2.291	1.706	2.833	<0.001

- We don't have sufficient evidence to say there is a significant difference between meals (p: 0.346).

- However, our best estimate of the meal effect is that changing the meal from BB to SS would decrease the number of oocysts in the midgut on average by a factor of $e^{\beta_{SS}} \rightarrow 0.761$. This means that the mean number of oocysts in the midgut is 0.761 times lower when the meal is SS than when the meal is BB with 95% confidence bounds [0.438,1.361].
- There is a significant generation effect. Generation F63 significantly differs from F8 (p: <0.001). This means that the mean number of oocysts in the midgut is 9.882 times higher in generation F63 than in F8, the 95% CI is [5.507,17.002].
- The plot below presents the observed oocysts counts by generation, along with oocyst count estimates and bootstrap 95% confidence intervals.

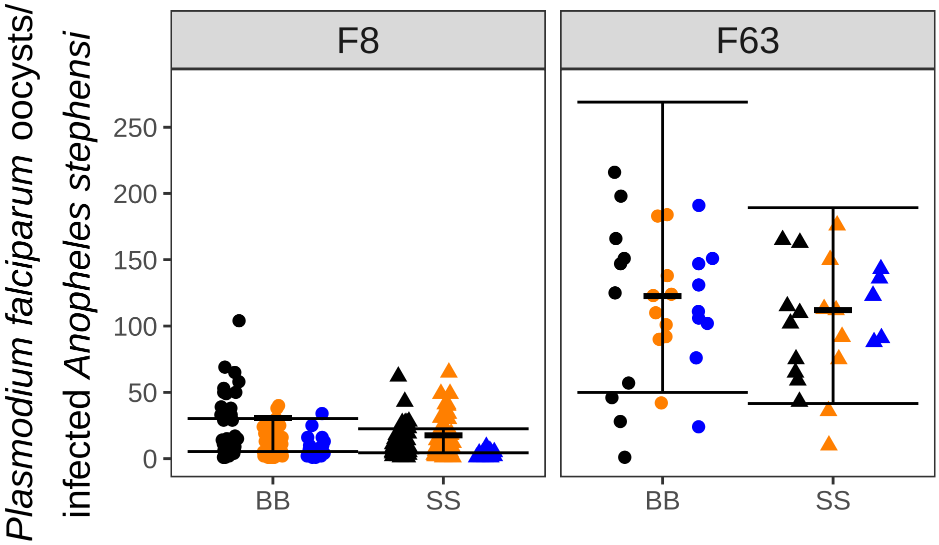

Figure 15: Observed (dots) and estimated (horizontal line) oocyst counts and bootstrap 95% confidence intervals (vertical lines) for *Anopheles stephensi*. Circles and triangles represent the data from the different replicate colonies fed on BB (circles), or SS (triangles), respectively. The colors correspond to the replicates which match the colors used in Additional file 3: Microsoft Excel workbook.

4.3.1 Vector Suceptibility Senstivity Analysis

Table 9: p-values from the quasi-binomial model comparing, for each generation, the proportion of dissected mosquitoes with oocysts from the two meal groups for *Anopheles stephensi*

	p-value
Generation,F8	0.621
Generation,F63	0.236

We found no significant differences in the fraction of infected mosquitoes at any of the three generations.

4.4 Summary Figure

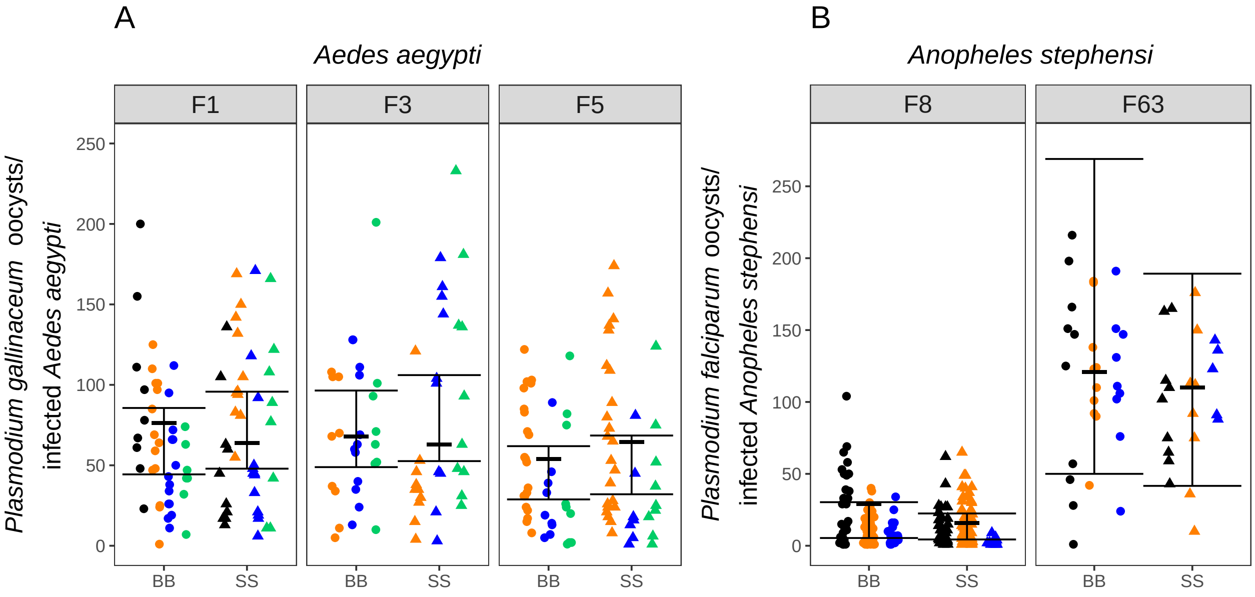

Figure 16: Oocyst counts from *Plasmodium* infections from colonies maintained on bovine blood (BB) or SkitoSnack (SS). A. Oocyst counts in mosquitoes Results from 3-4 biological replicate colonies of *Aedes aegypti* at generations F1, F3, and F5. B. Results from biological replicate colonies of *Anopheles stephensi* at generations F8, and F63. Colors represent the data from the different replicate colonies fed on BB (circles), or SS (triangles). Horizontal and vertical lines mark the estimated mean and bootstrap 95% confidence intervals of the oocyst counts.

5 Sporozoite Analysis

5.1 Analysis Details

- For *Aedes aegypti* we perform a two-sample t-test on log transformed data and transform the results back to represent the fold-change. For *Anopheles stephensi* we performed a generalized linear regression with a Gaussian family and fixed effects for meal and generation.
- Each considered data point represents a mosquito colony.
- The geometric mean ratio (GMR) is presented for SkitoSnack over bovine blood.

5.2 *Aedes aegypti*

- The geometric mean in the bovine blood group is 55010.19, and in the SkitoSnack group is 73139.27.
- The table below presents results for *Aedes aegypti*

Table 10: Geometric mean ratio (GMR) and 95% CI of sporozoite counts for *Aedes aegypti*

GMR	Lower 95%CI	Upper 95%CI	p.value
1.33	0.37	4.82	0.44

5.3 *Anopheles stephensi*

- The sporozoite means by generation and meal are presented in the table below:

Table 11: Sporozoite count geometric means by meal and generation for *Anopheles stephensi*

Generation	Geometric mean bovine blood	Geometric mean SkitoSnack
F8	4084.49	5004.48
F63	37379.72	51370.41

- The table below presents GMR of SS to BB along with 95% CI for *Anopheles stephensi*

Table 12: GMR and 95% CI of sporozoite counts for *Anopheles stephensi*

	GMR	Lower 95%CI	Upper 95%CI	p.value
SS vs BB	1.3	0.39	4.27	0.68
